# 2-D DIGE proteomic profiles of three strains of *Fusarium graminearum* grown in agmatine or glutamic acid medium

**DOI:** 10.1016/j.dib.2016.01.043

**Published:** 2016-01-29

**Authors:** Tommaso Serchi, Matias Pasquali, Céline C. Leclercq, Sébastien Planchon, Lucien Hoffmann, Jenny Renaut

**Affiliations:** Department of Environmental Research and Innovation, Luxembourg Institute of Science and Technology, 41, rue du Brill, L-4422 Belvaux, Luxembourg

**Keywords:** Comparative strain proteomics, Toxigenic fungi, Polyamines, Trichothecenes, Strain variability

## Abstract

2D DIGE proteomics data obtained from three strains belonging to *Fusarium graminearum* s.s. species growing in a glutamic acid or agmatine containing medium are provided.

A total of 381 protein species have been identified which do differ for abundance among the two treatments and among the strains (ANOVA<0.05 and abundance ratio>±1.3).

Data on the diversity of protein species profiles between the two media for each strain are made available. Shared profiles among strains are discussed in Pasquali et al. [1].

Here proteins that with diverse profile can be used to differentiate strains are highlighted. The full dataset allow to obtaining single strain proteomic profiles.

Specifications Table

**Table:** 

Subject area	*Biology*
More specific subject area	*Mycology*
Type of data	*Tables*
How data was acquired	*2-D Fluorescence Difference Gel Electrophoresis (2-D* DIGE*) and mass spectrometry*
Data format	*Analyzed data and raw gels*
Experimental factors	*Three different Fusarium graminearum strains belonging to three genetic chemotypes, medium nitrogen source*
Experimental features	*Comparative strain proteomic of 3 strains using 2-D DIGE. Fungal strains were grown in agmatine or glutamic acid containing medium for 8 days. Differentially abundant protein species between the 2 media and the 3 strains were identified.*
Data source location	*Luxembourg Institute of Science and Technology, Belvaux, Luxembourg Origin of the three strains used in the study are Luxembourg, Ohio (USA), Michigan (USA).*
Data accessibility	*The data are part of this article.*

Value of the data

• Level of diversity of the proteome of 3 different strains of *F. graminearum* in two growing conditions can be obtained from this dataset.
• Strain-dependent regulated protein species able to discriminate the three strains were identified.
• These data are useful to investigate strain specificity within *F. graminearum* species.

## 1 Data

**Fig. 1:**
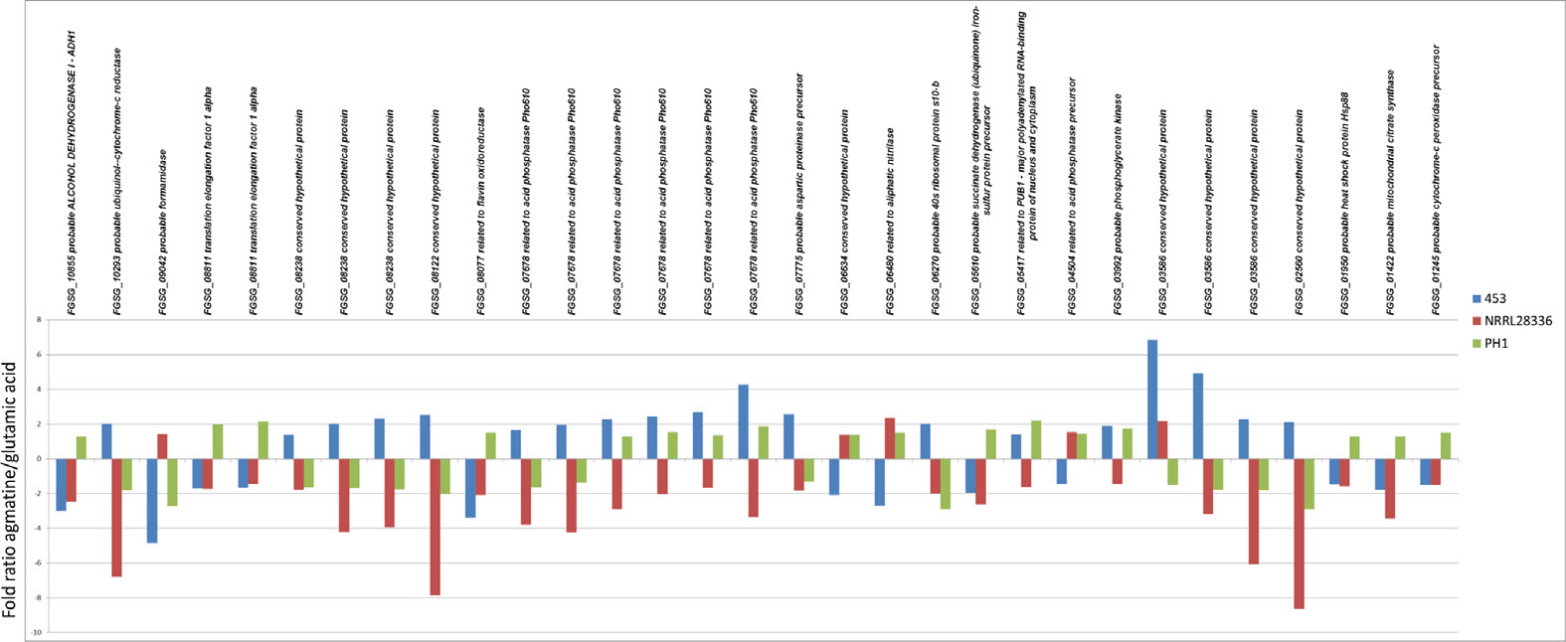
Expression ratio of protein species abundance between agmatine and glutamic acid media. Each FGSG line corresponds to a protein species that shows significant opposite behaviour among the three strains.

Agmatine and glutamic acid have a different effect on the phenotype of *F. graminearum* [1] that is also reflected in shared proteomic profiles discussed in [1]. Here we detail the complete list of all identified proteins that change significantly among strains or between conditions for each strain. In the list of protein species that are significantly shifting their abundance in each of the three strains representing *F. graminearum* toxigenic variability (Supplementary material 38) proteomic profiles for each strain can be obtained. By selecting the protein species with opposite behaviour among the strains we identified a subset of proteins that can be used to discriminate the three strains used here (Fig. 1). This dataset is also useful for comparing how different strains behave at the proteomic level when grown in agmatine or glutamic acid as the sole nitrogen source.

## 2 Experimental design, materials and methods

### 2.1 Growing conditions

Three *F. graminearum* strains (453, NRLL28336, PH1) differing for their geographic origin and genetic chemotypes [1], [2] were cultured in 2 toxin inducing media containing as the only nitrogen source glutamic acid or agmatine in four biological replicates. Briefly, the mycelium (as detailed in the video protocol by Pasquali et al. [3]) was incubated in Erlenmeyer flasks containing 30 g/L sucrose, 2.0 g/L glutamic acid (or 1.15 g/L agmatine), 1 g/L KH_2_PO_4_, 0.5 g/L MgSO_4_·7H_2_O, 0.5 KCl, 10 mg FeSO_4_·7H_2_O in 200 mL of trace elements solution (per 100 mL: 5 g KCl, 5 g ZnSO_4_·7H_2_O, 0.25 g CuSO_4_·5H_2_O, 50 mg MnSO_4_·H_2_O, 50 mg H_3_BO_3_, 50 mg NaMoO_4_·2H_2_O). Cultures were incubated in the dark, 150 rpm shaking at 22 °C for 8 days. The experiment was carried out with 4 biological replicates for each condition/strain. Ef-1alpha sequence of strain 453 was obtained following the protocol and the procedure described in [4], the other sequences were already available on NCBI.

### 2.2 Proteomics data collection and analysis

Full protein extraction was carried as described in [5]. Briefly, mycelia were ground with liquid nitrogen and extracted with ice-cold acetone containing 20% w/v trichloroacetic acid (TCA) and 1% w/v dithiothreitol. Proteins were let to precipitate overnight at −20 °C and then washed three times with ice-cold acetone. Resolubilization of the precipitated proteins was carried out in lysis buffer (7 M urea, 2 M thiourea, 4% w/v CHAPS, 30 mM Tris, pH 8.5) containing protease inhibitor mix (Roche) for 1 h on a rotary shaker at room temperature. The protein extracts were quantified using the Bradford method. 30 (thirty) μg of proteins for each sample (or internal standard) were labelled with 240 pmol of fluorochromes (CyDyes™, GE Healthcare) following the manufacturer’s instructions. Due to the presence of diverse pigmentation levels in the different strains, for the following labelling step, the samples were divided in 3 groups, each one representing one strain (4 biological replicates for each growing condition for each group giving a total of 8 samples for each group and 24 samples for the whole experiment). One internal standard was produced for each group. The four biological replicates were labelled using the dye swap technique: 2 replicates of the same growing condition were labelled with the Cy3 label and the other 2 replicates were labelled with the Cy5 label. A total of 12 gels were produced, each gel containing two biological replicates of the strains used and the respective internal standard, resulting in total protein load per gel of 90 μg. IPG buffer (Bio-Rad) and DeStreak reagent (GE Healthcare) were added to the mixed samples and internal standard prior the loading on the strip. Strips were passively rehydrated and proteins were loaded on 24 cm NL pH 3–10 IPG-strips (Bio-Rad) and isoelectric focusing (at 22 °C till approximately 100,000 Vh) was carried with IPG-phor system 3 (GE Healthcare). Strips were then equilibrated in equilibration solution with 1% w/v DTT for 15 min and then 2.5% w/v iodoacetamide for 15 min. The second dimension was carried out with 12.5% polyacrylamide pre-cast gels (Gelcompany) following manufacturer’s instructions. Images were acquired using a Typhoon9400 (GE Healthcare). Thirty six raw gels images (Supplementary material 2–37) were obtained and analysed by DeCyder v.7.0 software (GE Healthcare). After confirming lack of preferential labelling, exclusion filters and manual detection of spots were applied to each gel in order to obtain the most representative gel image. Gels were exported to the biological variation analysis (BVA) module. Twenty spots were manually landmarked to allow the software to perform inter-gel matching. Extensive manual spot matching was then done to ensure correct matching of spots. The EDA module allowed linking, standardizing and comparing the different groups for the subsequent statistical analysis. Spots considered to be consistent and reproducible (at least present in 75% of biological replicates and with 1-way ANOVA≤0.05) were subjected to statistical analysis. Within the same *F. graminearum* strain, mycelia grown in the presence of agmatine were pairwise compared to those grown in the presence of glutamic acid: spots resulting in a difference of at least ±30% and with a *p*-value (*T*-test) ≤0.05 were considered as spots of interest and selected for subsequent picking and protein identification. In order to compare the effects of the strain and medium and their interaction, 2-way ANOVA multivariate analysis was performed. Moreover also spots resulting in a *p*-value≤0.05 in at least one among 2-way ANOVA *Fusarium* strain, 2-way ANOVA medium or 2-way ANOVA interaction were added to the list of the spots of interest and selected for the subsequent picking and protein identification.

Spots were picked from the gel mainly from the 453 map [5] and few random verifications were carried out on the two master gels of the other experimental groups. All picked spots were then digested by trypsin for 6 h at 37 °C using an Ettan Dalt Spot Handling Workstation (GE Healthcare) before acquisition of peptide mass spectra with a 4800 MALDI-TOF-TOF analyzer (ABSciex). One MS spectrum accumulating 1500 laser shots in total was acquired and the highest 8 precursors, having a signal-to-noise ratio of more than 30, were automatically selected for subsequent MS/MS analysis. The MIPS *F. graminearum* database v 3.2 [6] was used for Mascot analysis (matrix Science) using a combined approach of protein mass finger print and MS/MS.

Complete NCBI proteins database check was also performed on all unknown proteins. All searches were carried out using a mass tolerance window of 150 ppm for protein mass fingerprint and 0.75 Da for the MS/MS analysis of fragments. Up to two trypsin missed cleavages were considered as acceptable. The search parameters allowed for carbamidomethylation of cysteine (fixed modification), oxidation of methionine as well as oxidation of tryptophan, tryptophan to kynurenine and double oxidation of tryptophan to N-formylkynurenine (as variable modifications). Only identifications with a Mascot *p*-value ≤0.05 were considered and manually checked and validated. Significance threshold for the combined MOWSE score was ≥54, while for fragmented peptides the significance threshold was ≥30.

## Footnotes

Appendix A Supplementary data associated with this article can be found in the online version at doi:10.1016/j.dib.2016.01.043.
