# Supplementary material for: 2-D DIGE proteomic profiles of three strains of Fusarium graminearum grown in agmatine or glutamic acid medium
Source: Data Brief. 2016 Jan 29;6:985–8. doi: 10.1016/j.dib.2016.01.043 (PMC4778272; doi:10.1016/j.dib.2016.01.043)
Supplement: Supplementary file 1 — Supplementary material [file mmc1.doc]

**Mmc1.** Legend for supplementary files: mmc2-37 includes 2D-DIGE gels of *F. graminearum* strains (strain 453, strain NRRL28336 and strain PH1). Raw, un-processed gels are reported. For each gel, the Cy3 and Cy5 channels (experimental gels) are reported, as well as the Cy2 channel (internal standard). Mmc 38 indicates the protein profiles of the 3 strains with abundance values. Mmc 39 is the conflict of interest document.

**Mmc2.** Supplementary file mmc2 refers to Strain 453 in Glutamic acid - replicate 1 (file mmc2.zip – G1G1 HS Cy3)

**Mmc3.** Supplementary file mmc3 refers to Strain 453 in Agmatine acid - replicate 1 (file mmc3.zip – G1G1 HS Cy5)

**Mmc4.** Supplementary file mmc4 refers to Strain 453 - Internal standard for replicate 1 (file mmc4.zip – G1G1 HS STANDARD CY2)

**Mmc5.** Supplementary file mmc5 refers to Strain 453 in Glutamic acid - replicate 2 (file mmc5.zip – G1G2 rZ Cy3)

**Mmc6.** Supplementary file mmc6 refers to Strain 453 in Agmatine - replicate 2 (file mmc6.zip – G1G2 rZ Cy5)

**Mmc7.** Supplementary file mmc7 refers to Strain 453 - Internal standard for replicate 2 (file mmc7.zip – G1G2 rZ STANDARD CY2)

**Mmc8.** Supplementary file mmc8 refers to Strain 453 in Glutamic acid - replicate 3 (file mmc8.zip – G1G3 r1 Cy5)

**Mmc9.** Supplementary file mmc9 refers to Strain 453 - Internal standard for replicate 3 (file mmc9.zip – G1G3 r1 STANDARD CY2)

**Mmc10.** Supplementary file mmc10 refers to Strain 453 in Agmatine - replicate 3 (file mmc10.zip – G1G3 rZ Cy3)

**Mmc11.** Supplementary file mmc11 refers to Strain 453 in Agmatine - replicate 4 (file mmc11.zip – G1G4 sD Cy3)

**Mmc12.** Supplementary file mmc12 refers to Strain 453 in Glutamic acid - replicate 4 (file mmc12.zip – G1G4 sD Cy5)

**Mmc13.** Supplementary file mmc13 refers to Strain 453 - Internal standard for replicate 4 (file mmc13.zip – G1G4 sD STANDARD CY2)

**Mmc14.** Supplementary file mmc14 refers to Strain NRRL28336 in Glutamic acid - replicate 1 (file mmc14.zip – G2G1 sL Cy3)

**Mmc15.** Supplementary file mmc15 refers to Strain NRRL28336 in Agmatine - replicate 1 (file mmc15.zip – G2G1 sL Cy5)

**Mmc16.** Supplementary file mmc16 refers to Strain NRRL28336 - Internal standard for replicate 1 (file mmc16.zip – G2G1 sL STANDARD CY2)

**Mmc17.** Supplementary file mmc17 refers to Strain NRRL28336 in Glutamic acid - replicate 2 (file mmc17.zip – G2G2 sf Cy3)

**Mmc18.** Supplementary file mmc18 refers to Strain NRRL28336 in Agmatine - replicate 2 (file mmc18.zip – G2G2 sf Cy5)

**Mmc19.** Supplementary file mmc19 refers to Strain NRRL28336 - Internal standard for replicate 2 (file mmc19.zip – G2G2 sf STANDARD CY2)

**Mmc20.** Supplementary file mmc20 refers to Strain NRRL28336 in Agmatine - replicate 3 (file mmc20.zip – G2G3 ss Cy3)

**Mmc21.** Supplementary file 21 refers to Strain NRRL28336 in Glutamic acid - replicate 3 (file mmc21.zip – G2G3 ss Cy5)

**Mmc22.** Supplementary file mmc22 refers to Strain NRRL28336 - Internal standard for replicate 3 (file mmc22.zip – G2G3 ss STANDARD CY2)

**Mmc23.** Supplementary file mmc23 refers to Strain NRRL28336 in Agmatine - replicate 4 (file mmc23.zip – G2G4 sK Cy3)

**Mmc24.** Supplementary file mmc24 refers to Strain NRRL28336 in Glutamic acid - replicate 4 (file mmc24.zip – G2G4 sK Cy5)

**Mmc25.** Supplementary file mmc25 refers to Strain NRRL28336 - Internal standard for replicate 4 (file mmc25.zip – G2G4 sK STANDARD CY2)

**Mmc26.** Supplementary file mmc26 refers to Strain PH1 in Glutamic acid - replicate 1 (file mmc26.zip – G3G1 sk Cy3)

**Mmc27.** Supplementary file mmc27 refers to Strain PH1 in Agmatine - replicate 1 (file mmc27.zip – G3G1 sk Cy5)

**Mmc28.** Supplementary file mmc28 refers to Strain PH1 - Internal standard for replicate 1 (file mmc28.zip – G3G1 sk STANDARD CY2)

**Mmc29.** Supplementary file mmc29 refers to Strain PH1 in Glutamic acid - replicate 2 (file mmc29.zip – G3G2 sC Cy3)

**Mmc30.** Supplementary file mmc30 refers to Strain PH1 in Agmatine - replicate 2 (file mmc30.zip – G3G2 sC Cy5)

**Mmc31.** Supplementary file mmc31 refers to Strain PH1 - Internal standard for replicate 2 (file mmc31.zip – G3G2 sC STANDARD CY2)

**Mmc32.** Supplementary file mmc32 refers to Strain PH1 in Agmatine - replicate 3 (file mmc32.zip – G3G3 sG Cy3)

**Mmc33.** Supplementary file mmc33 refers to Strain PH1 in Glutamic acid - replicate 3 (file mmc33.zip – G3G3 sG Cy5)

**Mmc34.** Supplementary file mmc34 refers to Strain PH1 - Internal standard for replicate 3 (file mmc34.zip – G3G3 sG STANDARD CY2)

**Mmc35.** Supplementary file mmc35 refers to Strain PH1 in Agmatine - replicate 4 (file mmc35.zip – G3G4 rL Cy3)

**Mmc36.** Supplementary file mmc36 refers to Strain PH1 in Glutamic acid - replicate 4 (file mmc36.zip – G3G4 rL Cy5)

**Mmc37.** Supplementary file mmc37 refers to Strain PH1 - Internal standard for replicate 4 (file mmc37.zip – G3G4 rL STANDARD CY2)

**Mmc38.** Supplementary file mmc38 with 3 excel sheets summarizing the proteomic profiles of the three strains used in this work.

**Mmc39.** Supplementary file mmc39 is the conflict of interest document signed by all co-authors.
