# Supplementary figures and images for: 2-D DIGE proteomic profiles of three strains of Fusarium graminearum grown in agmatine or glutamic acid medium
Source: Data Brief. 2016 Jan 29;6:985–8. doi: 10.1016/j.dib.2016.01.043 (PMC4778272; doi:10.1016/j.dib.2016.01.043)

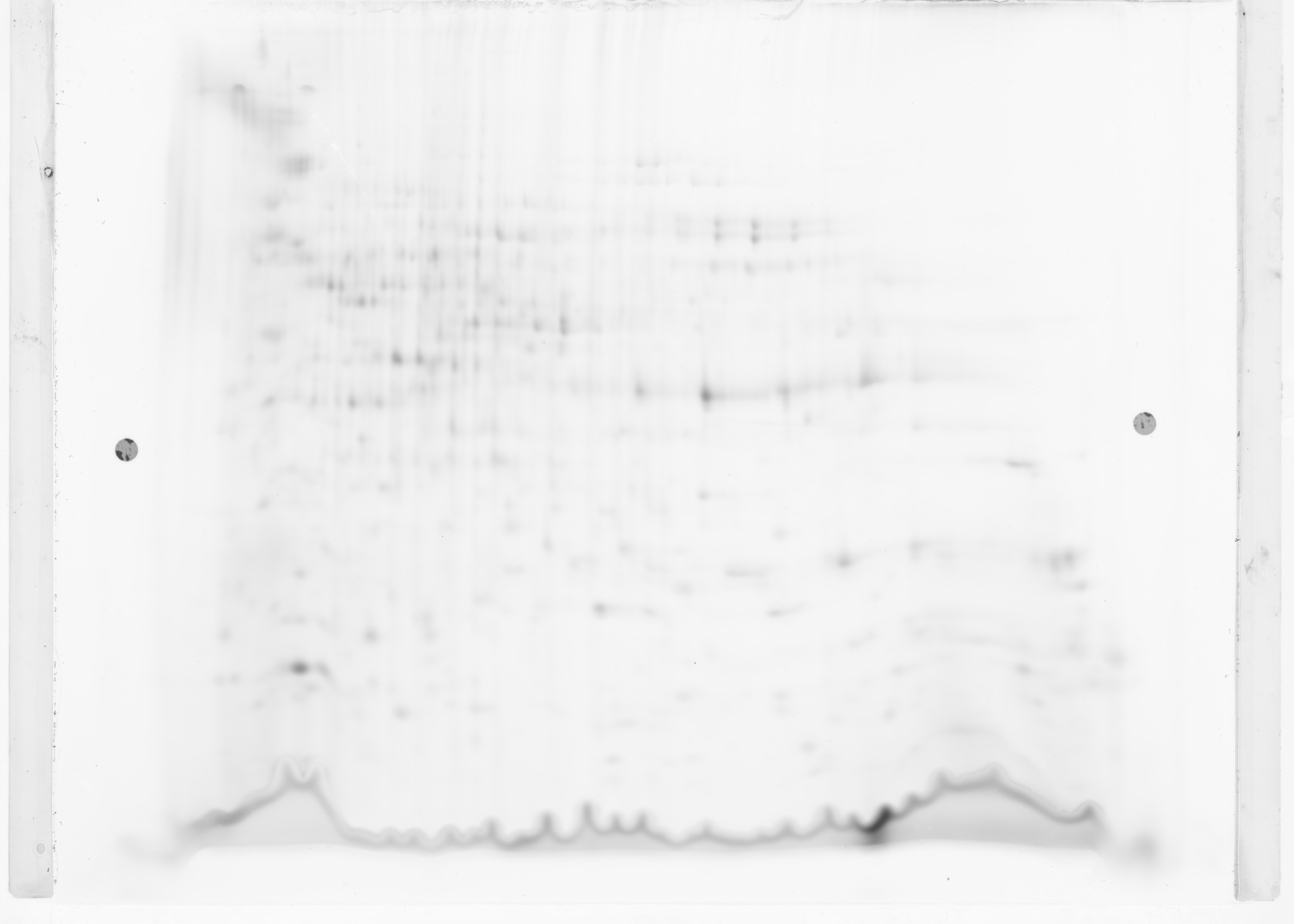

Supplement: Supplementary file 2 — Supplementary material [file mmc2.zip › mmc2.gel]

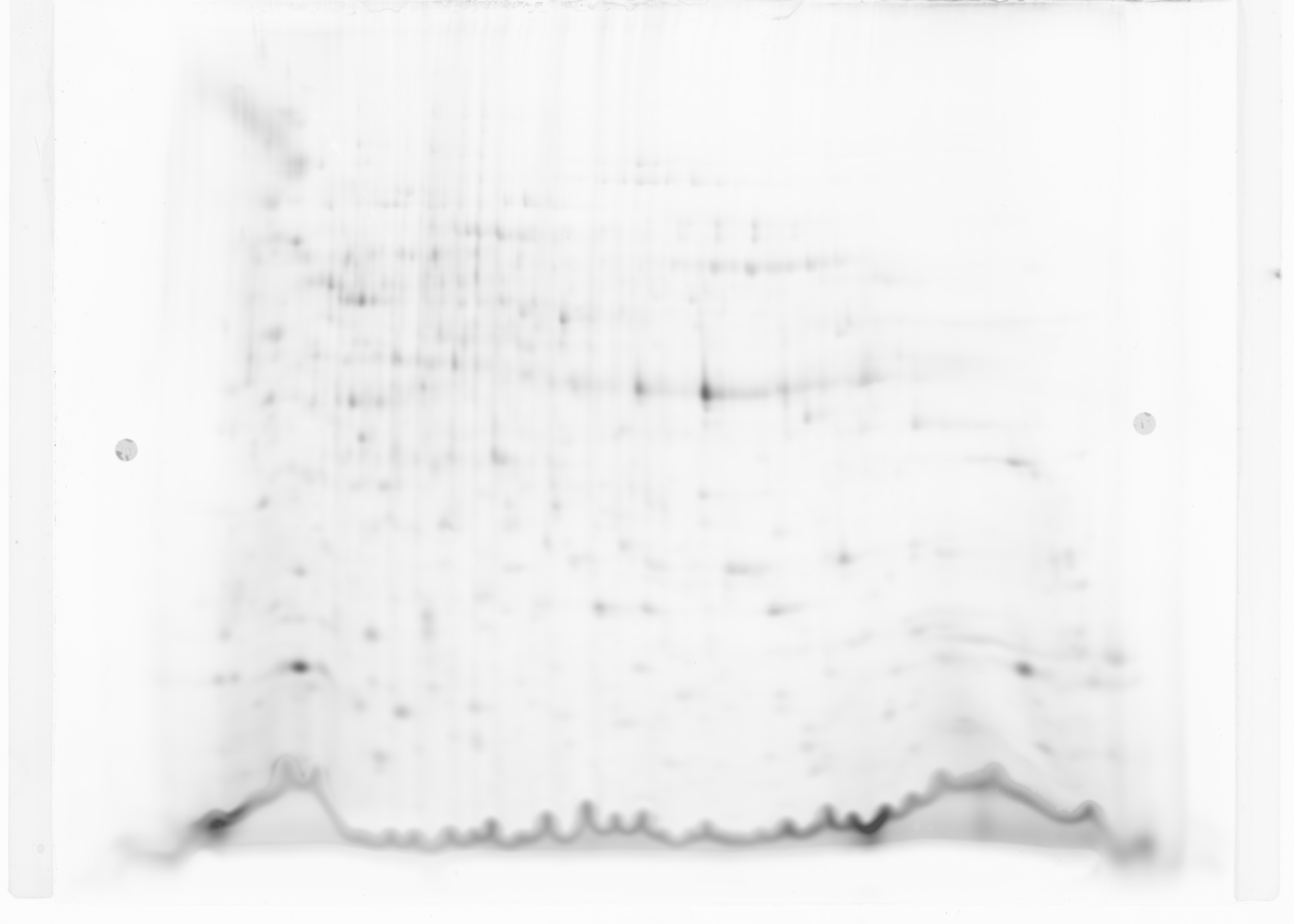

Supplement: Supplementary file 3 — Supplementary material [file mmc3.zip › mmc3.gel]

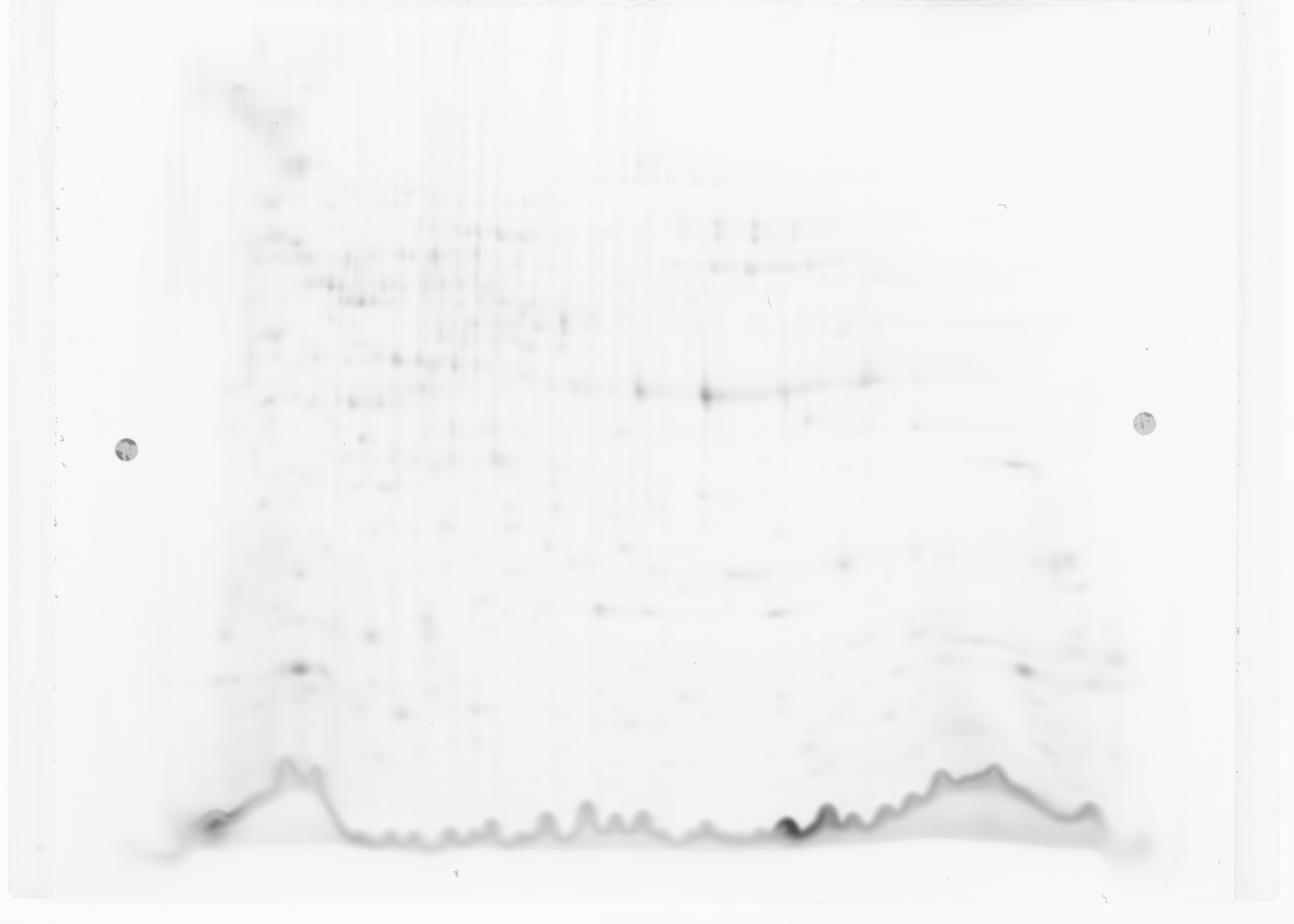

Supplement: Supplementary file 4 — Supplementary material [file mmc4.zip › mmc4.gel]

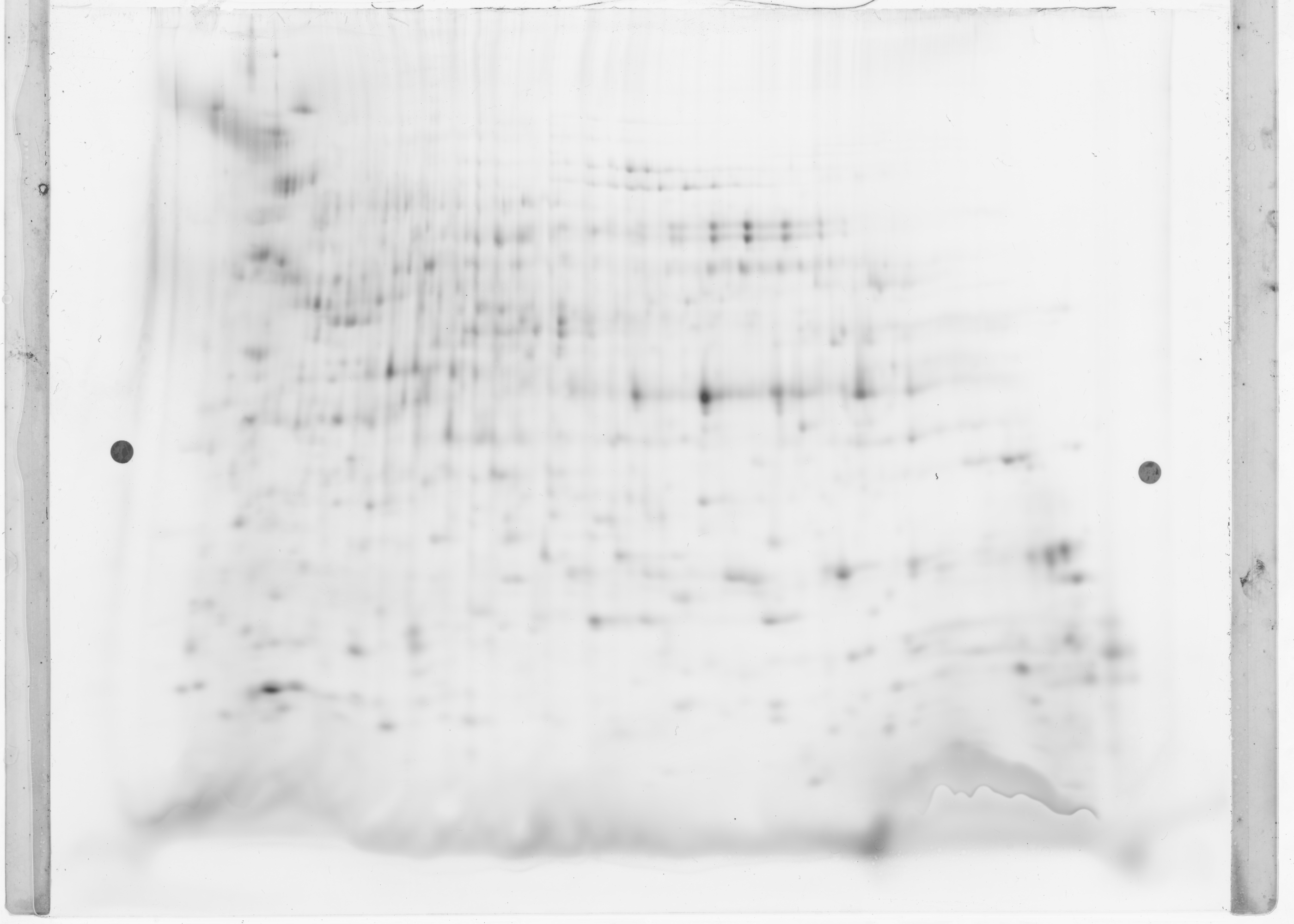

Supplement: Supplementary file 5 — Supplementary material [file mmc5.zip › mmc5.gel]

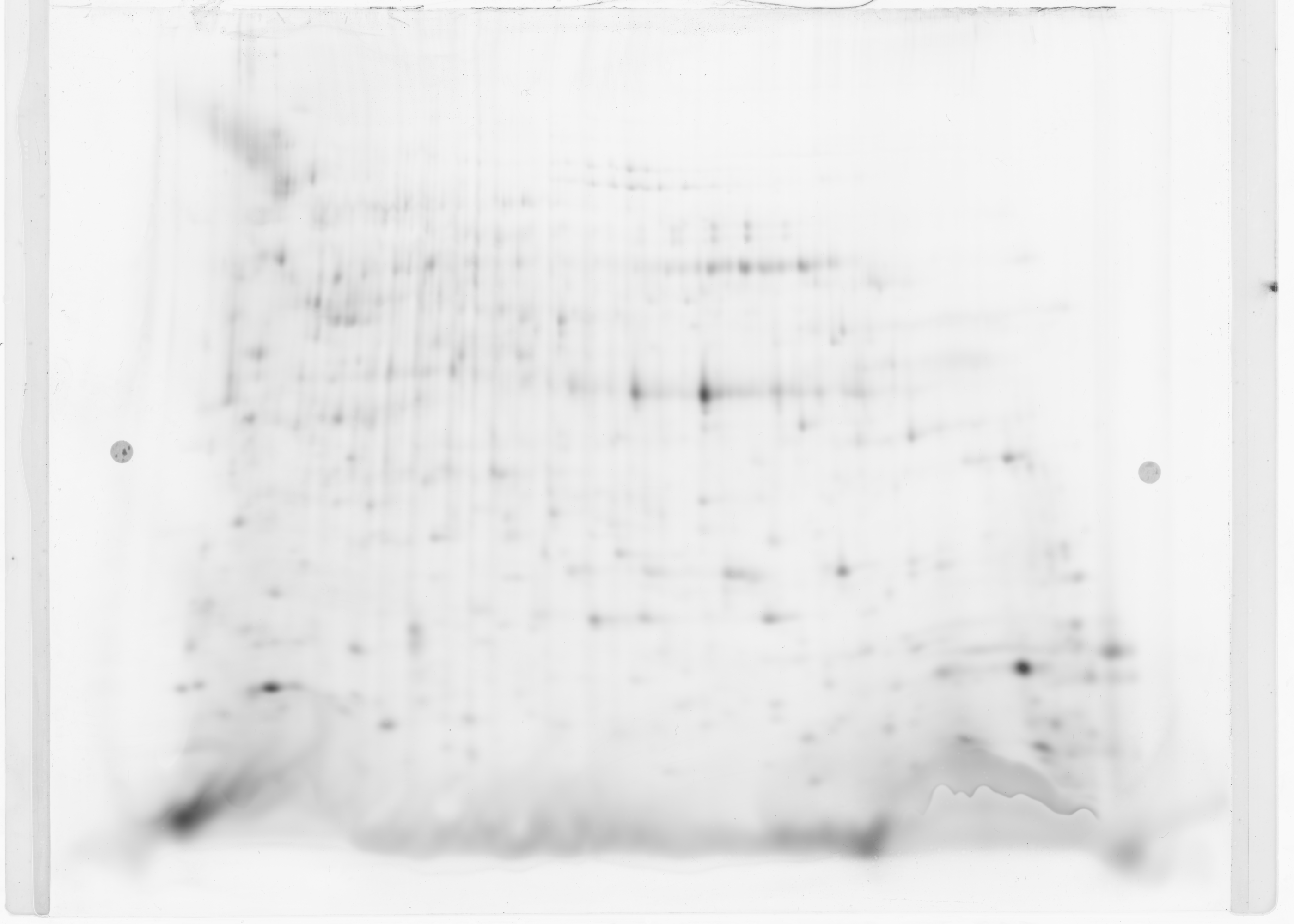

Supplement: Supplementary file 6 — Supplementary material [file mmc6.zip › mmc6.gel]

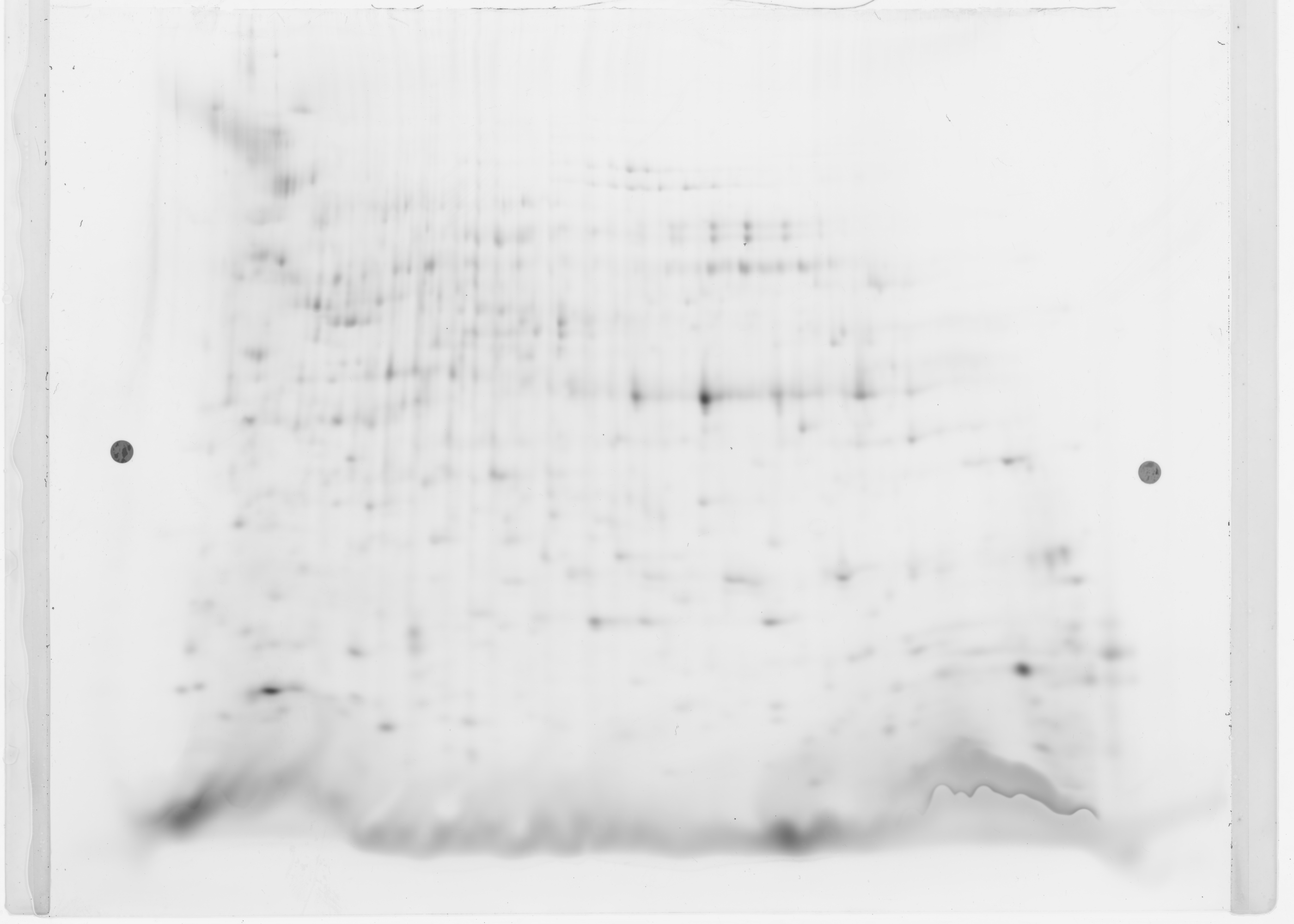

Supplement: Supplementary file 7 — Supplementary material [file mmc7.zip › mmc7.gel]

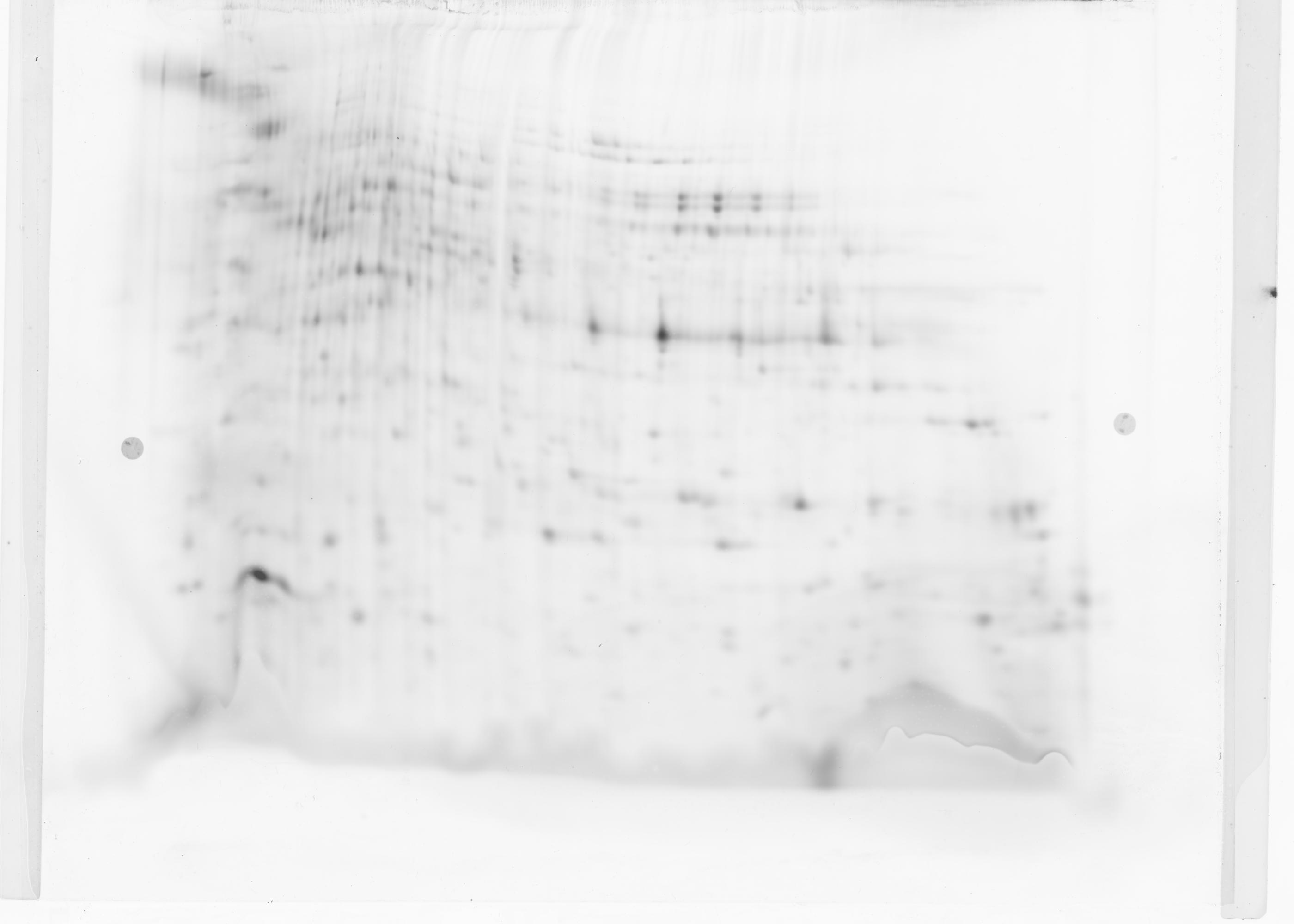

Supplement: Supplementary file 8 — Supplementary material [file mmc8.zip › mmc8.gel]

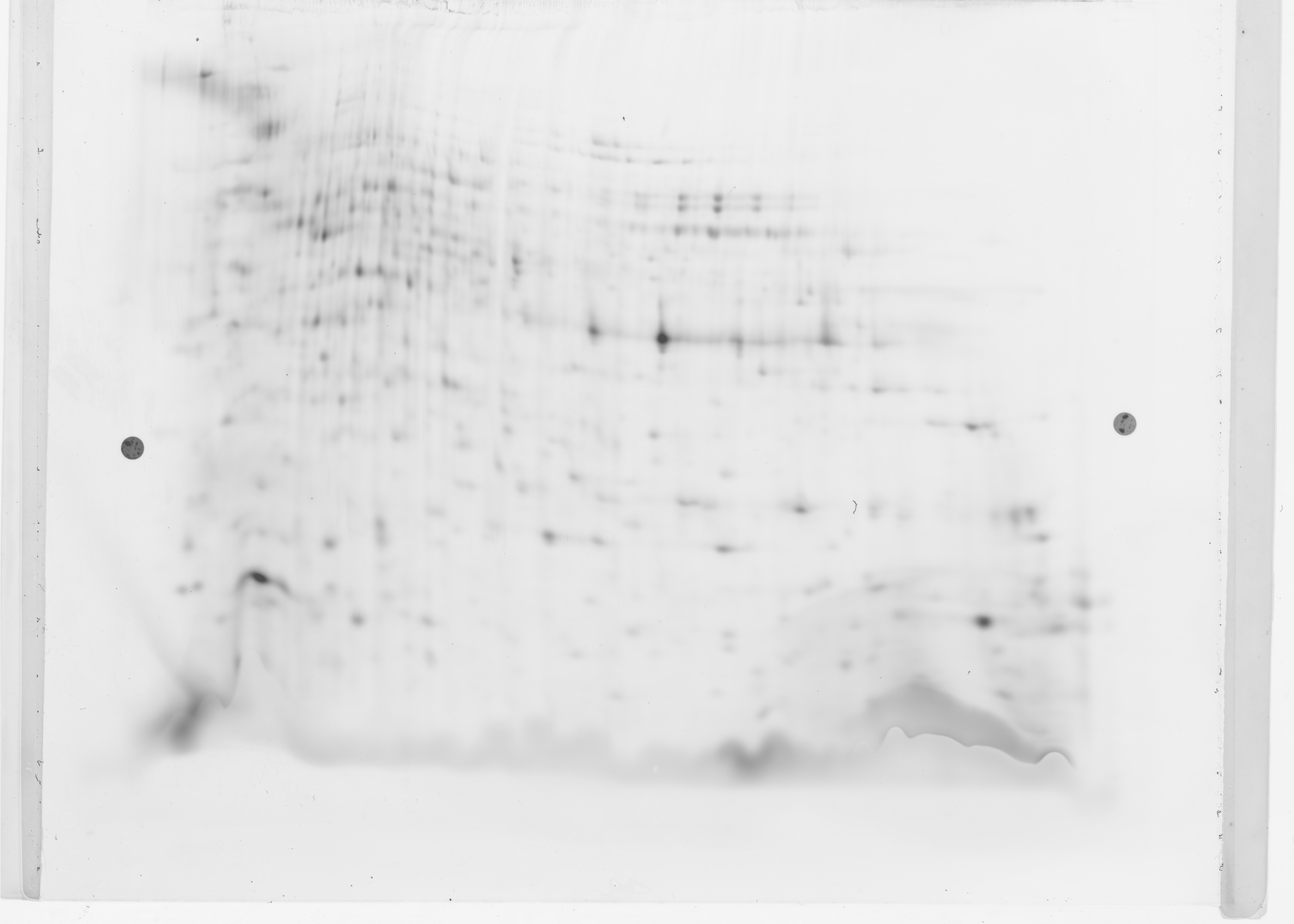

Supplement: Supplementary file 9 — Supplementary material [file mmc9.zip › mmc9.gel]

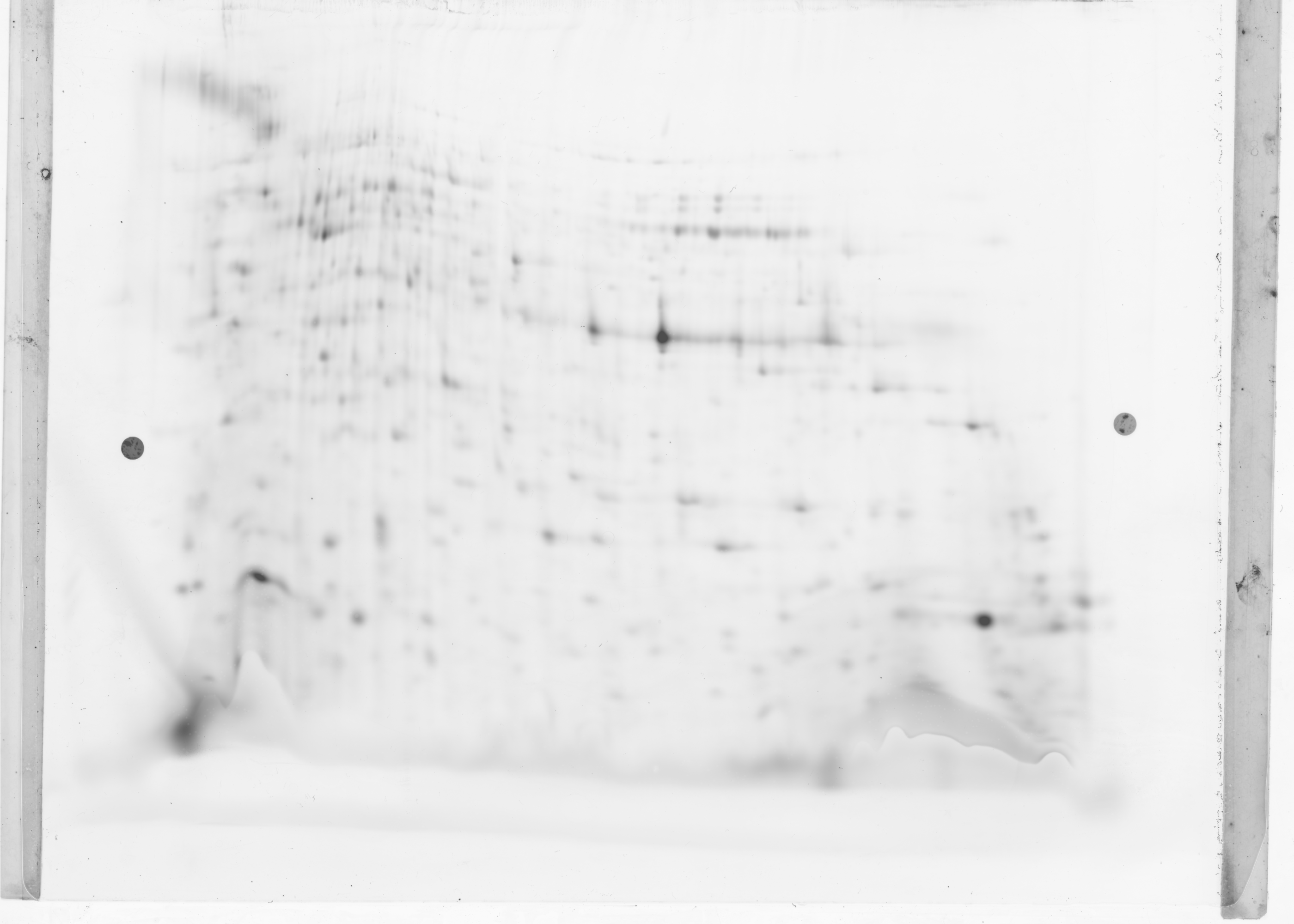

Supplement: Supplementary file 10 — Supplementary material [file mmc10.zip › mmc10.gel]

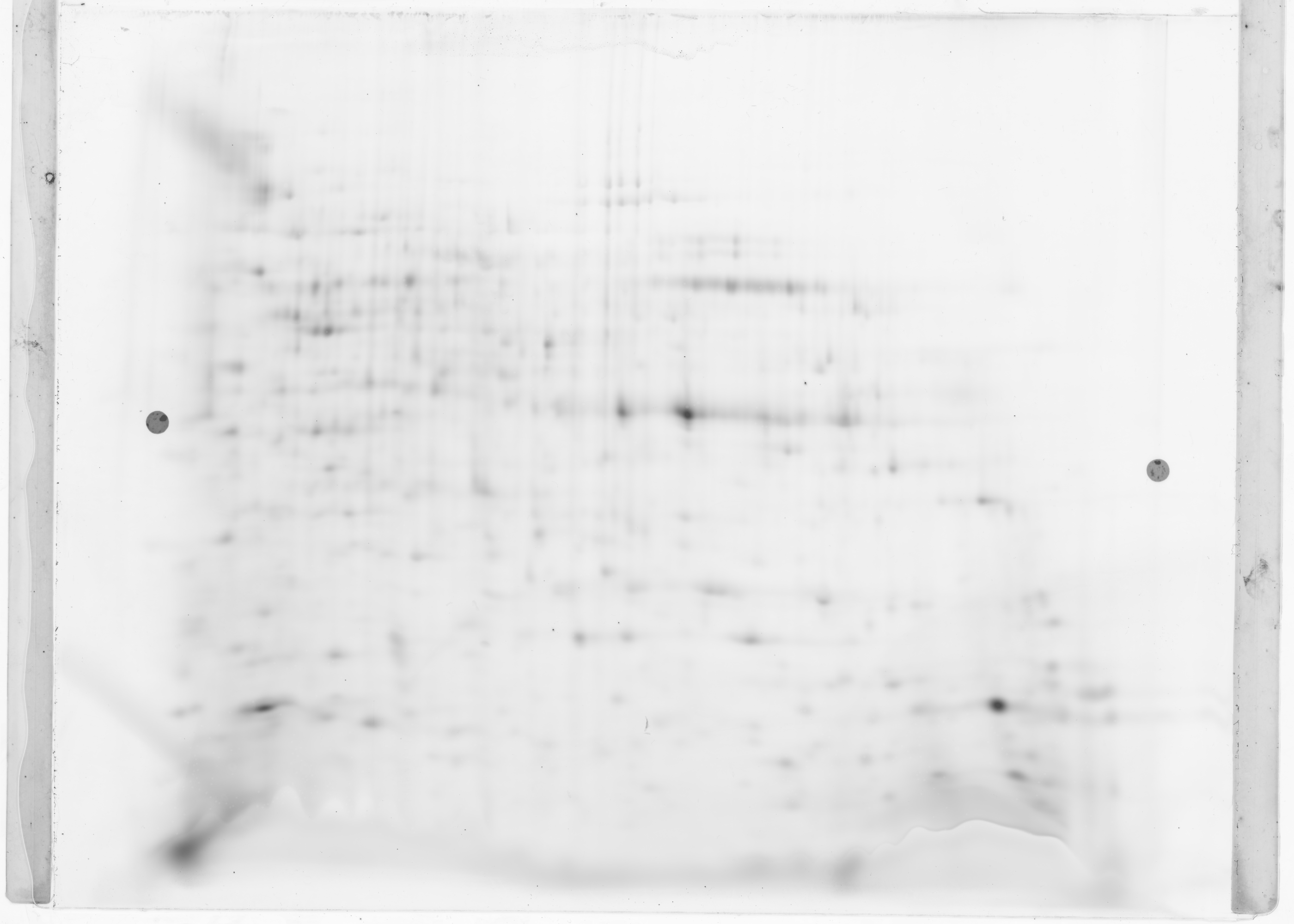

Supplement: Supplementary file 11 — Supplementary material [file mmc11.zip › mmc11.gel]

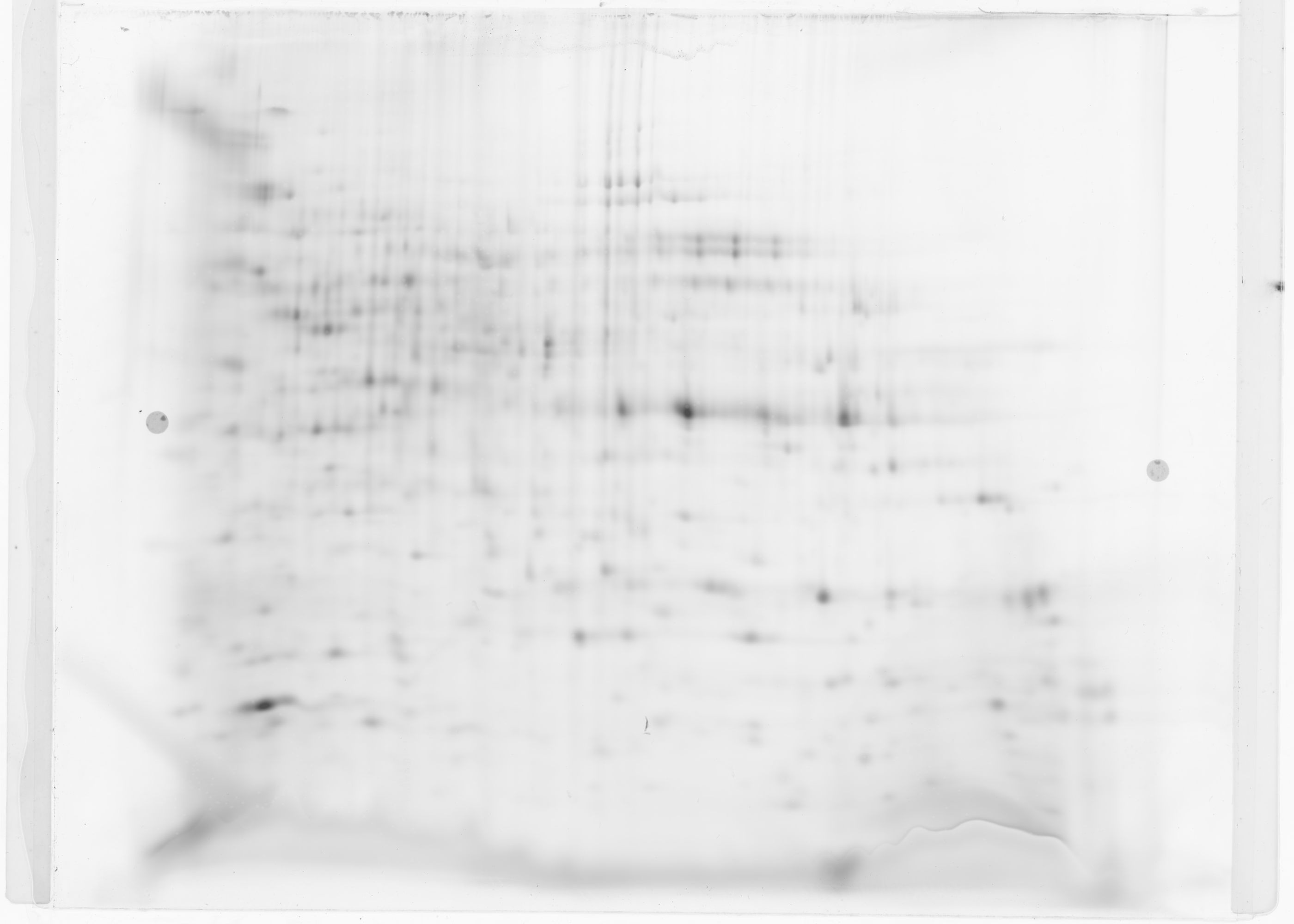

Supplement: Supplementary file 12 — Supplementary material [file mmc12.zip › mmc12.gel]

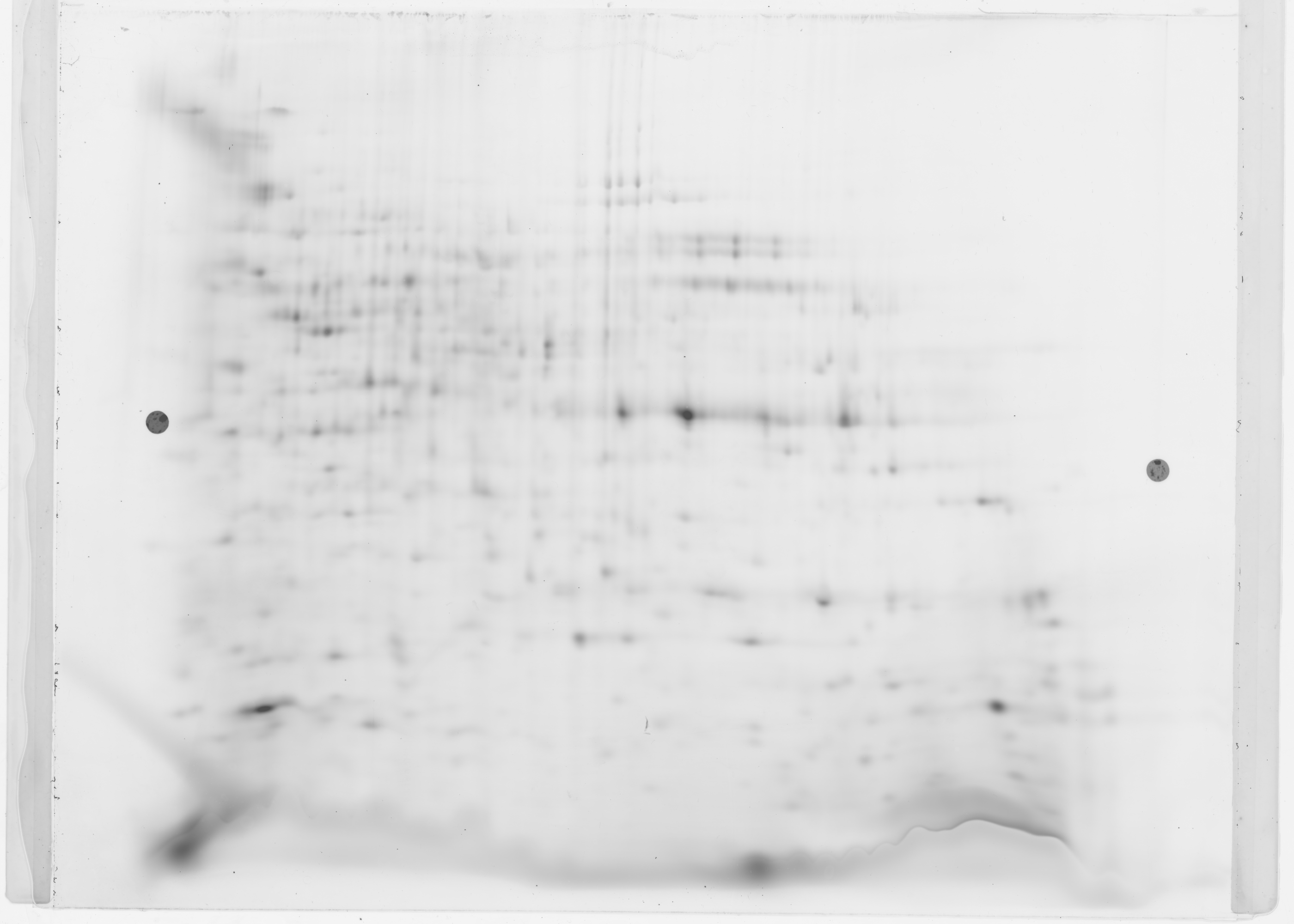

Supplement: Supplementary file 13 — Supplementary material [file mmc13.zip › mmc13.gel]

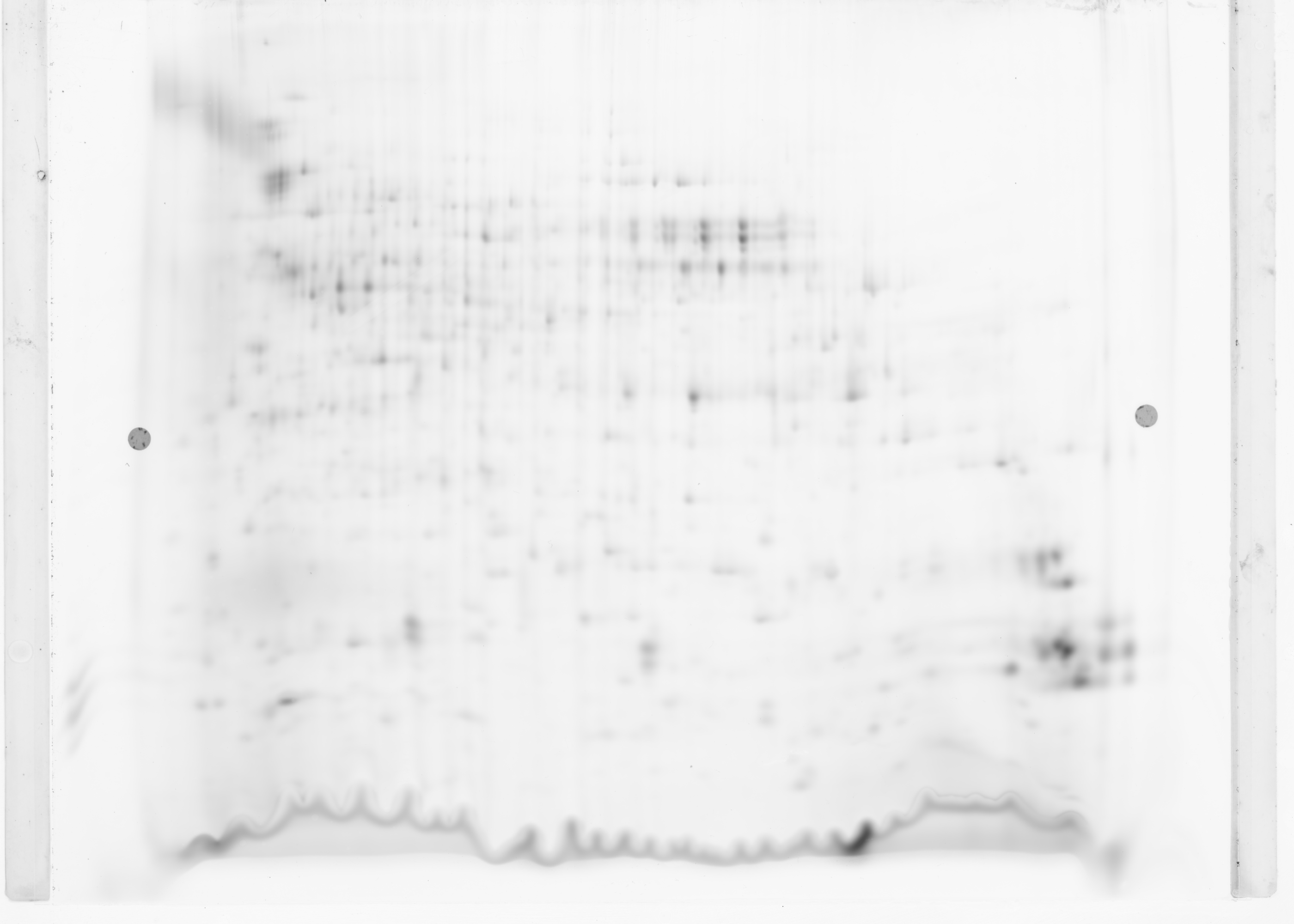

Supplement: Supplementary file 14 — Supplementary material [file mmc14.zip › mmc14.gel]

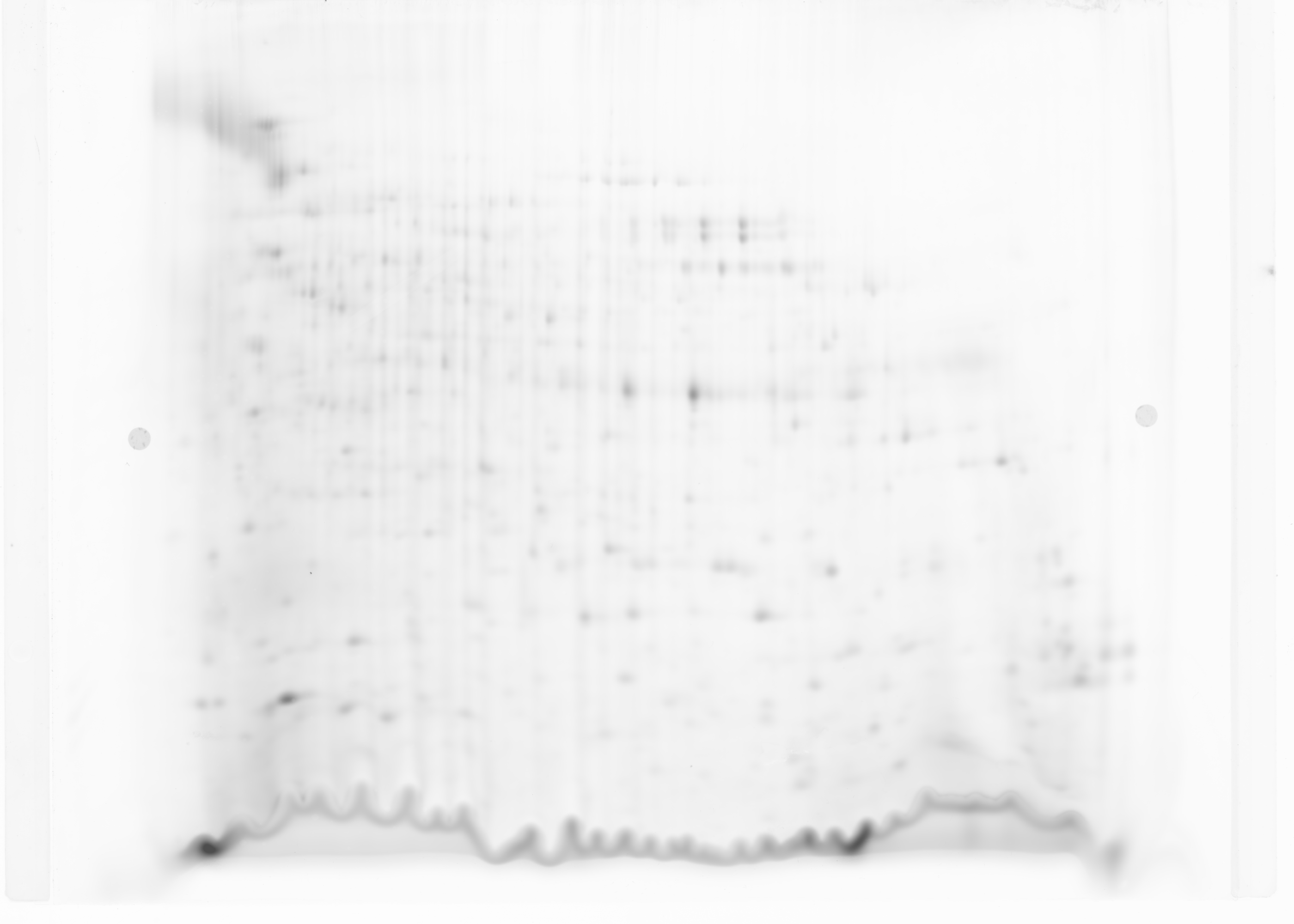

Supplement: Supplementary file 15 — Supplementary material [file mmc15.zip › mmc15.gel]

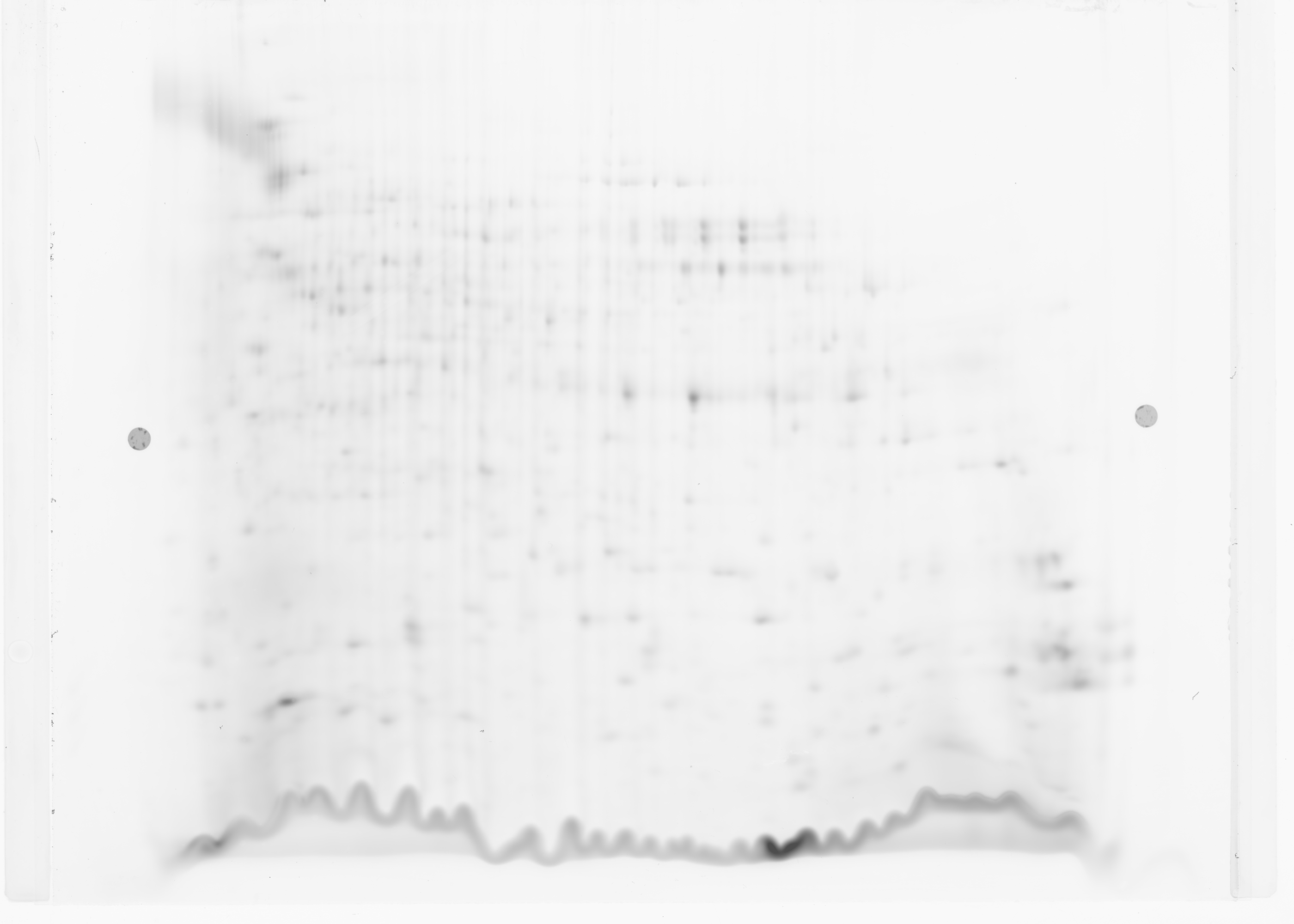

Supplement: Supplementary file 16 — Supplementary material [file mmc16.zip › mmc16.gel]

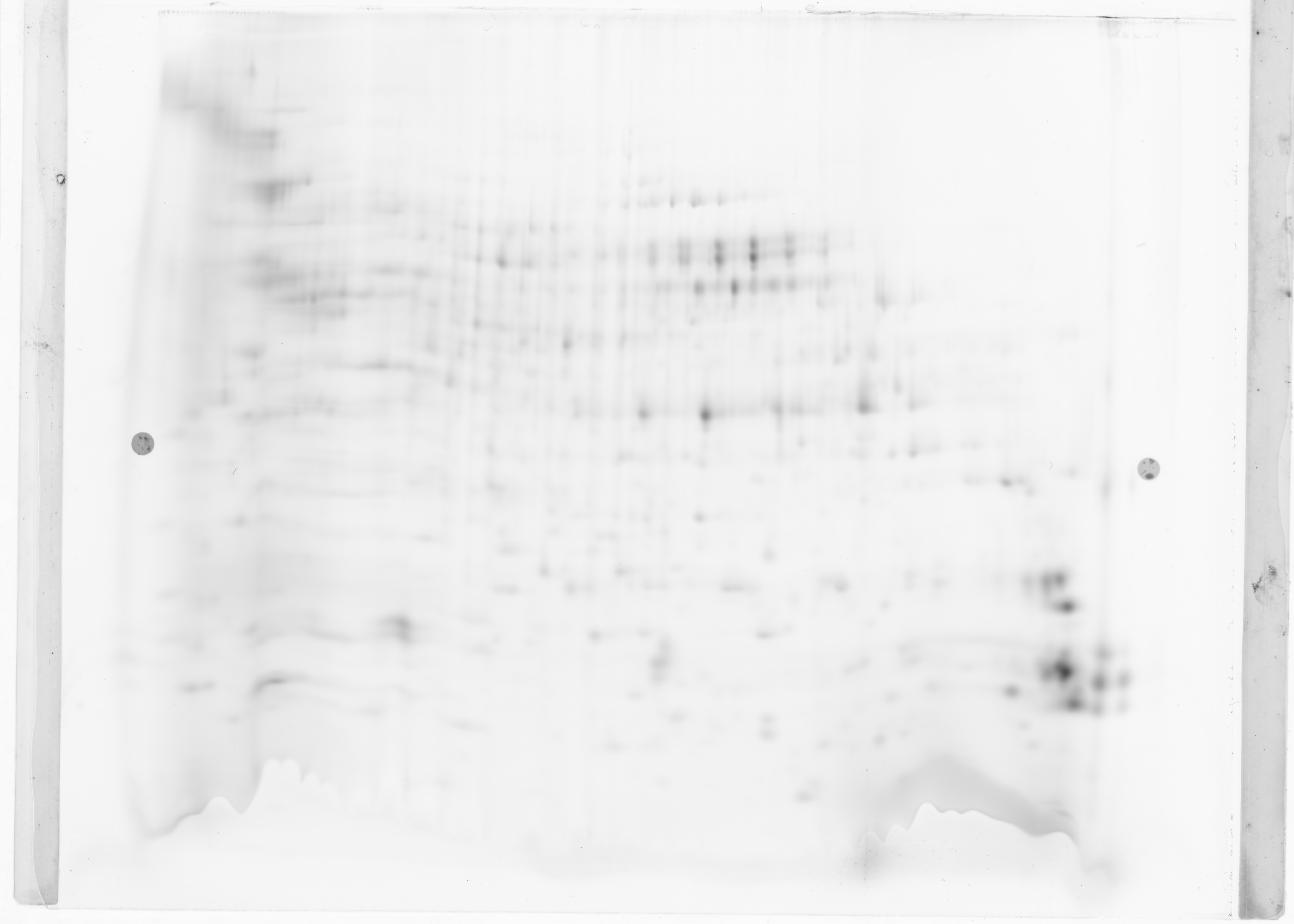

Supplement: Supplementary file 17 — Supplementary material [file mmc17.zip › mmc17.gel]

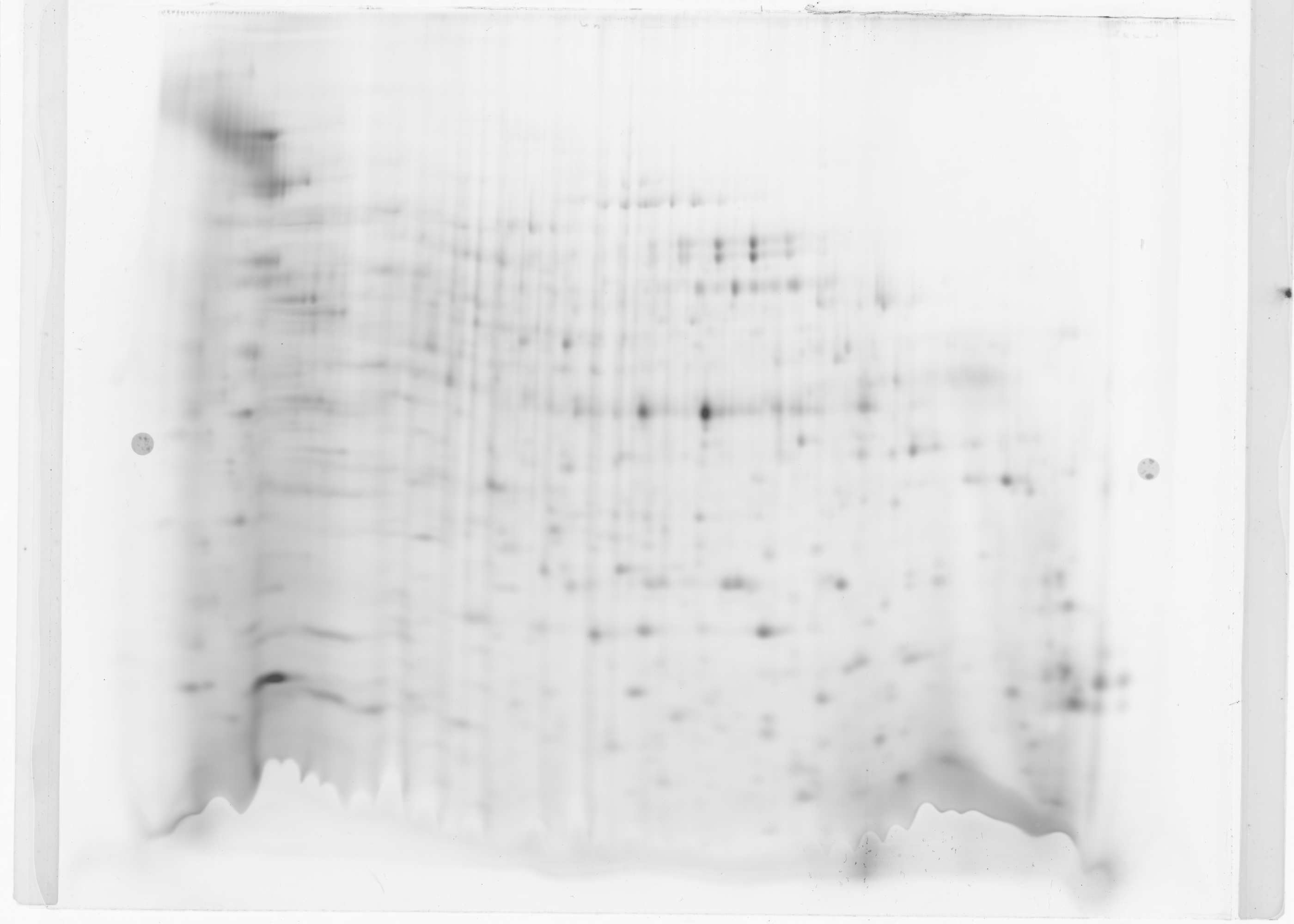

Supplement: Supplementary file 18 — Supplementary material [file mmc18.zip › mmc18.gel]

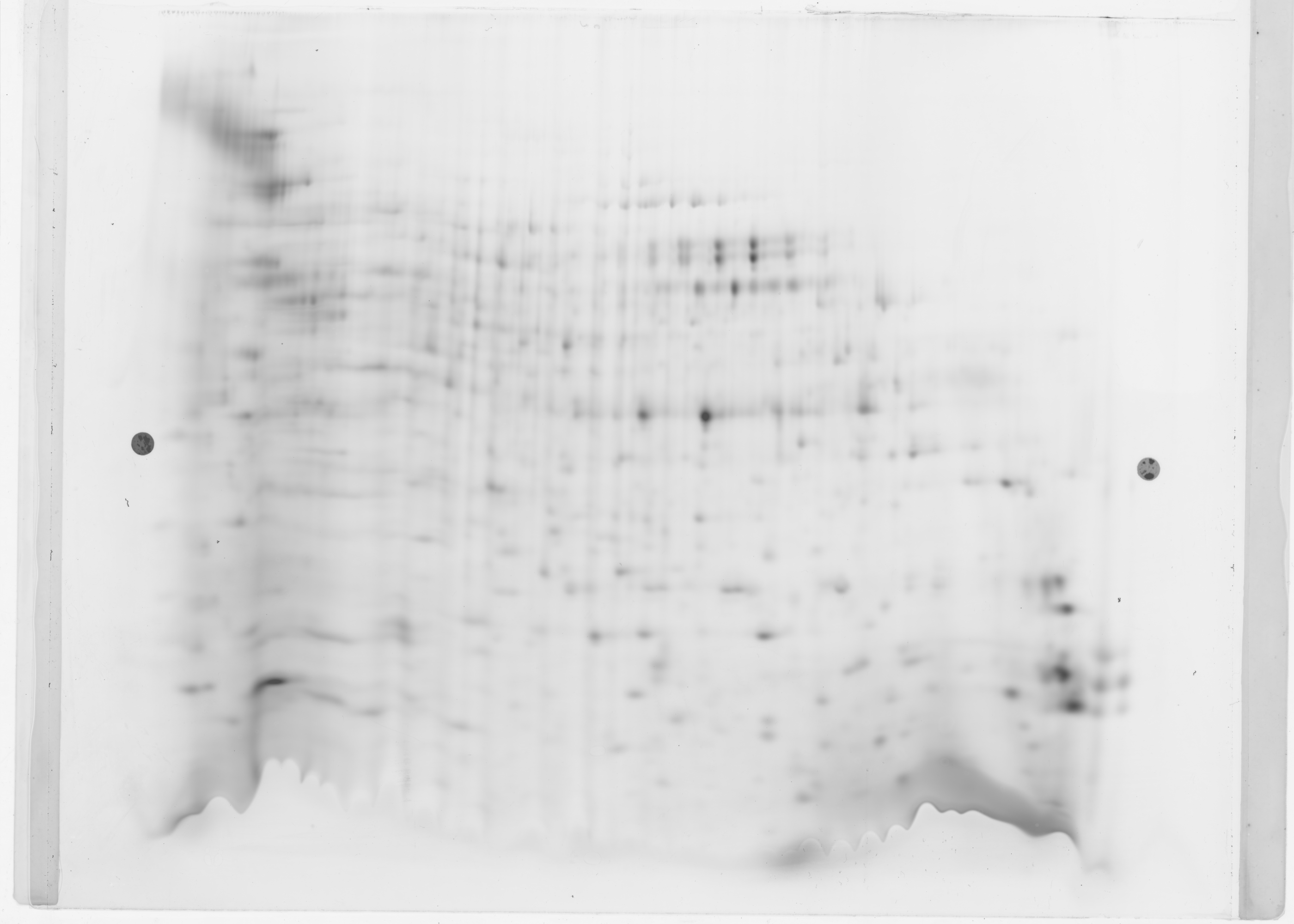

Supplement: Supplementary file 19 — Supplementary material [file mmc19.zip › mmc19.gel]

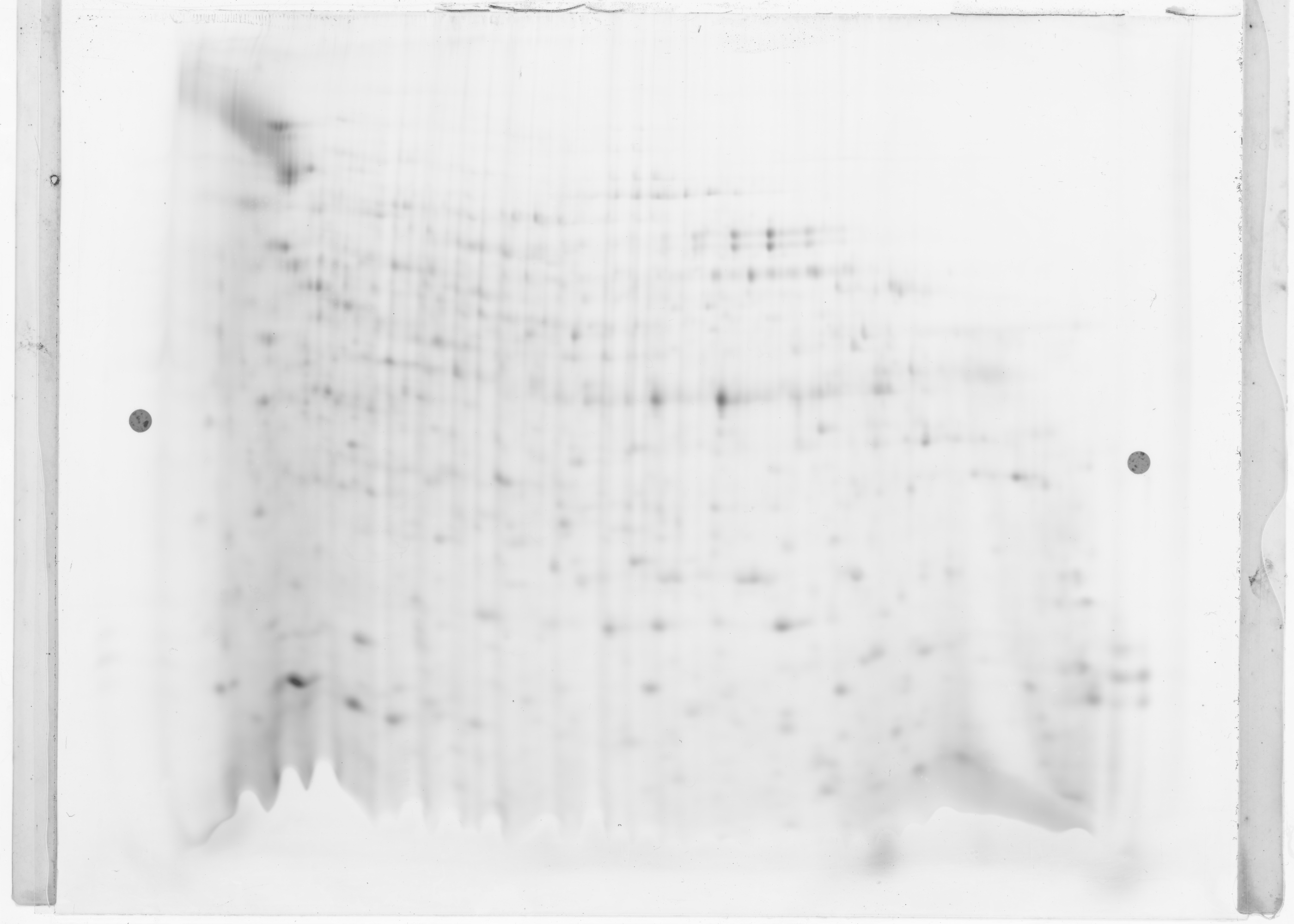

Supplement: Supplementary file 20 — Supplementary material [file mmc20.zip › mmc20.gel]

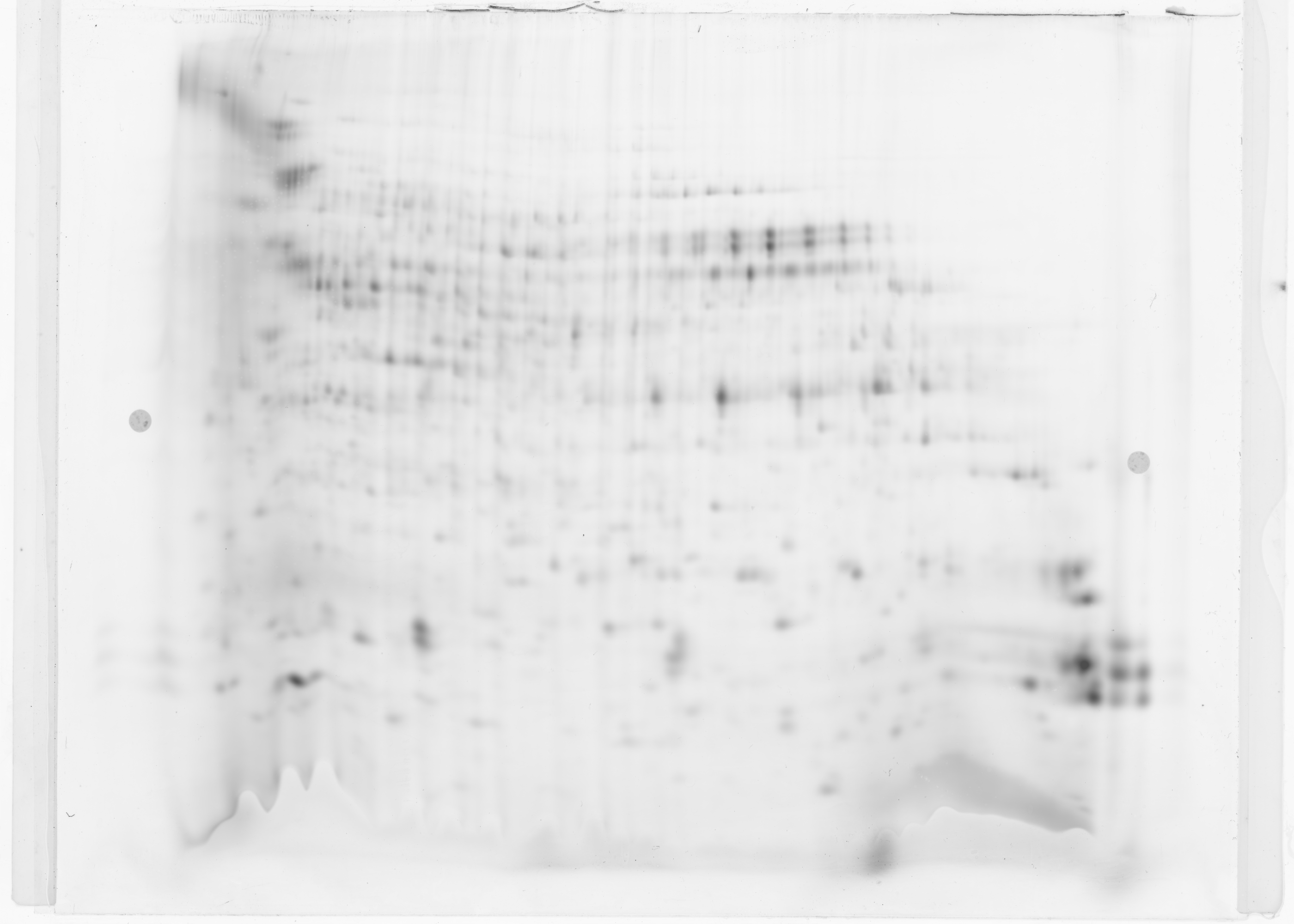

Supplement: Supplementary file 21 — Supplementary material [file mmc21.zip › mmc21.gel]

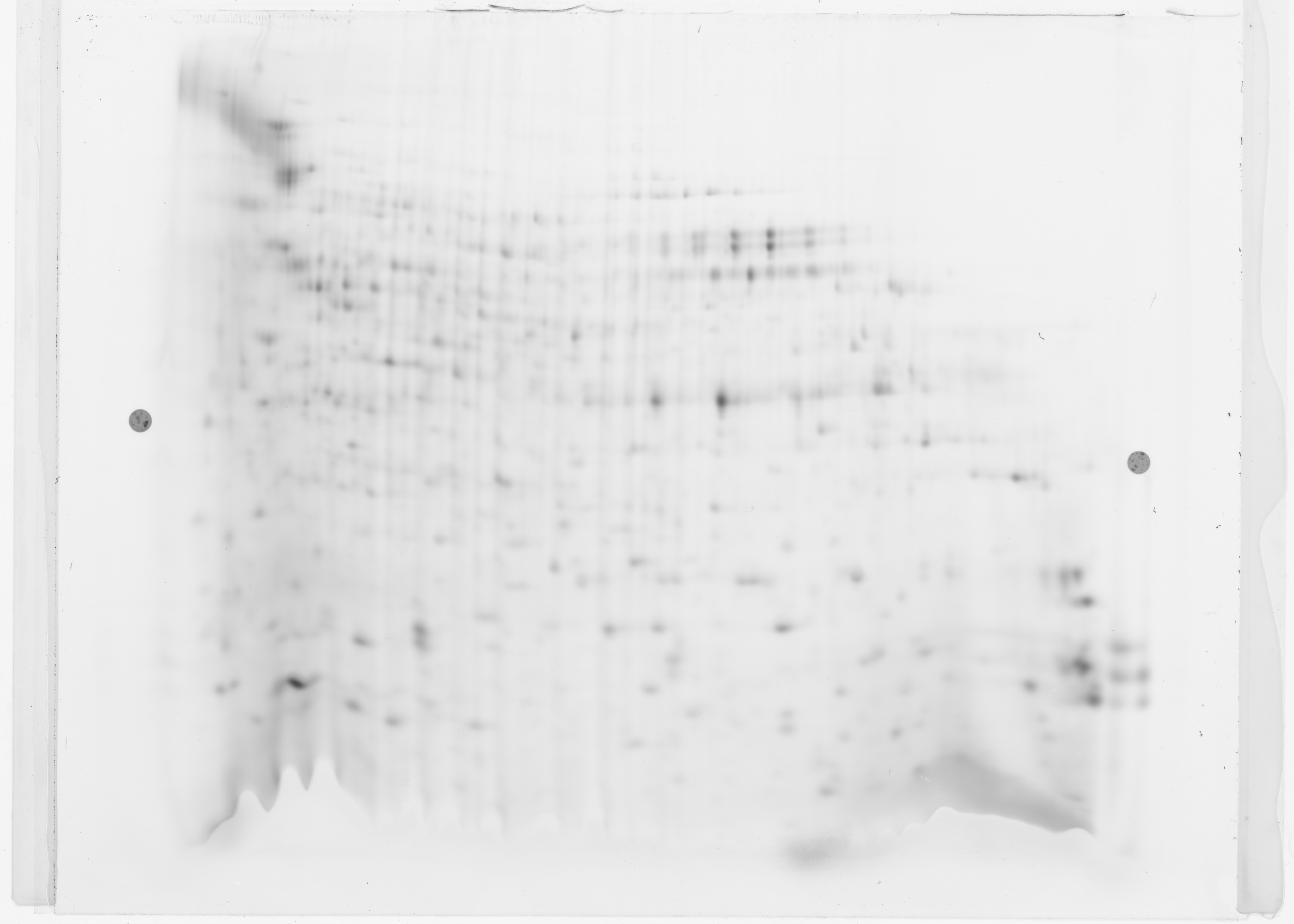

Supplement: Supplementary file 22 — Supplementary material [file mmc22.zip › mmc22.gel]

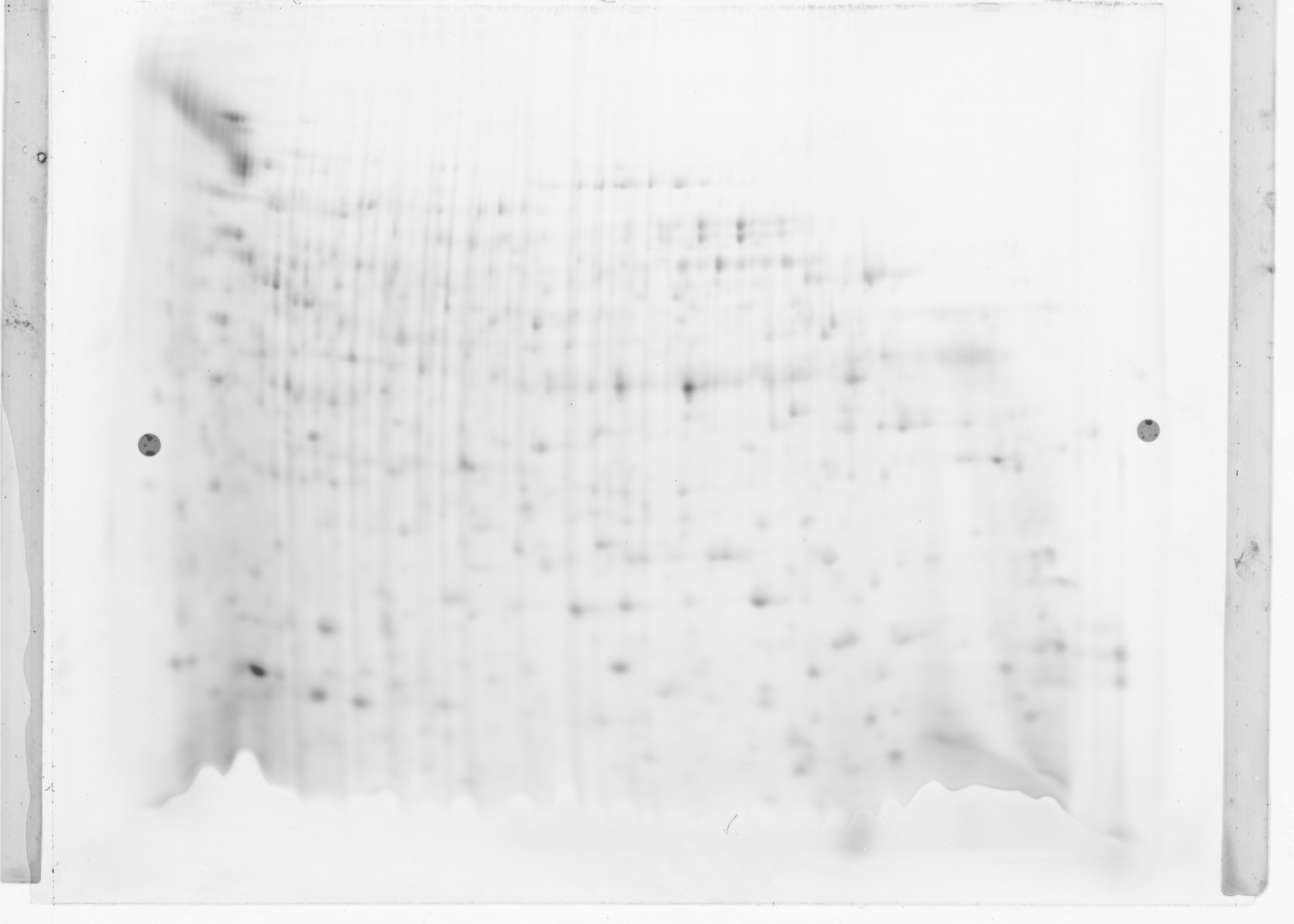

Supplement: Supplementary file 23 — Supplementary material [file mmc23.zip › mmc23.gel]

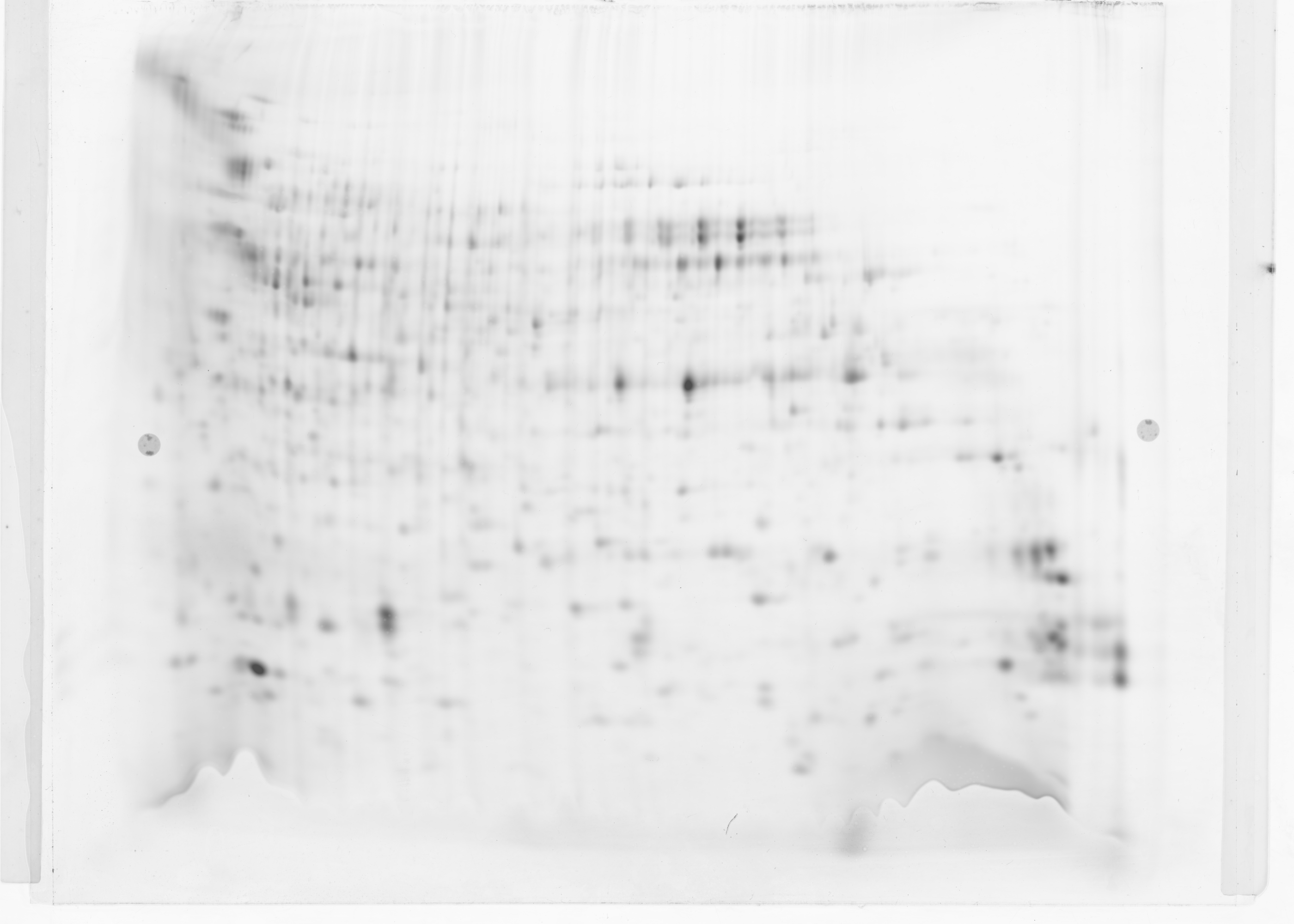

Supplement: Supplementary file 24 — Supplementary material [file mmc24.zip › mmc24.gel]

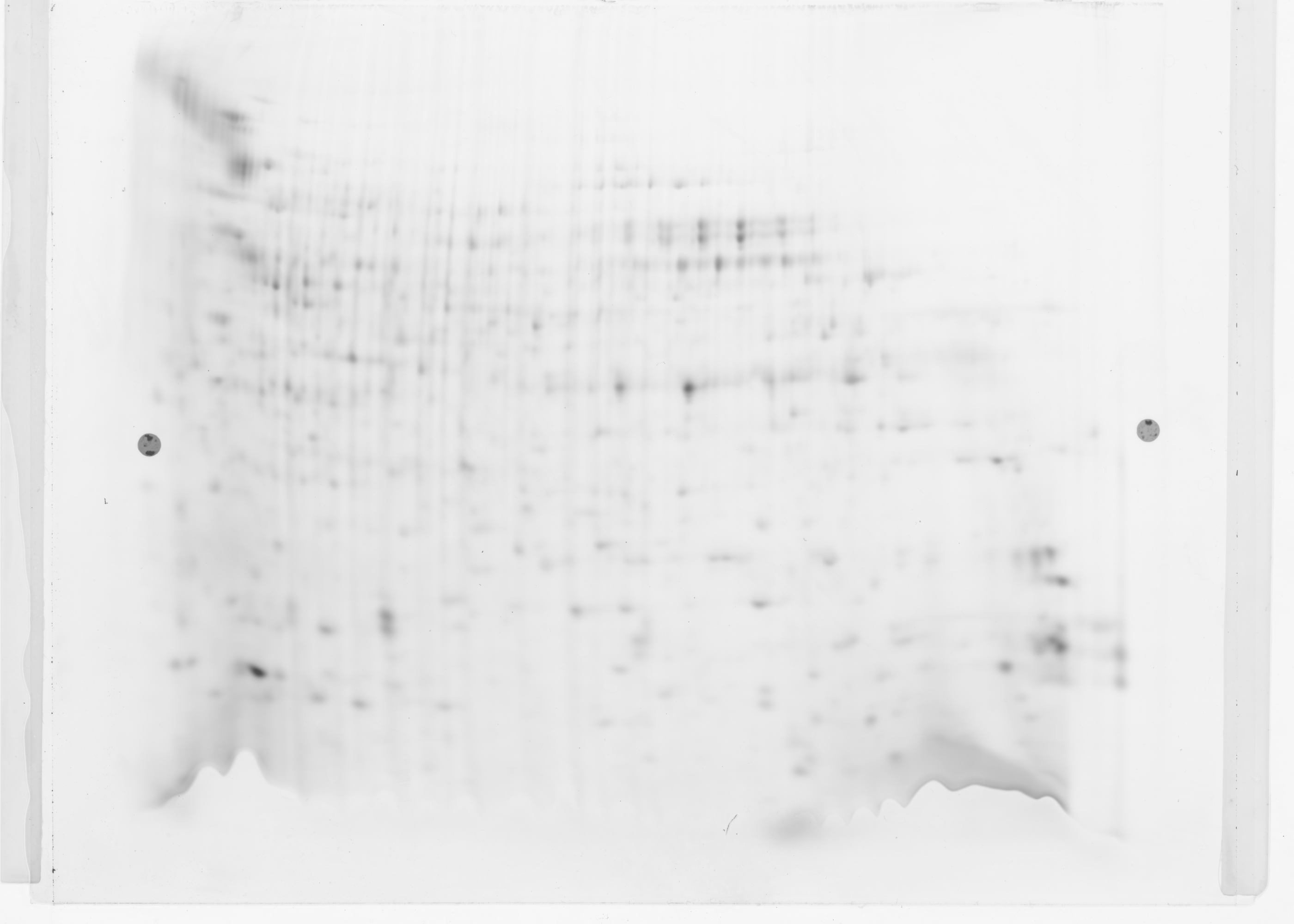

Supplement: Supplementary file 25 — Supplementary material [file mmc25.zip › mmc25.gel]

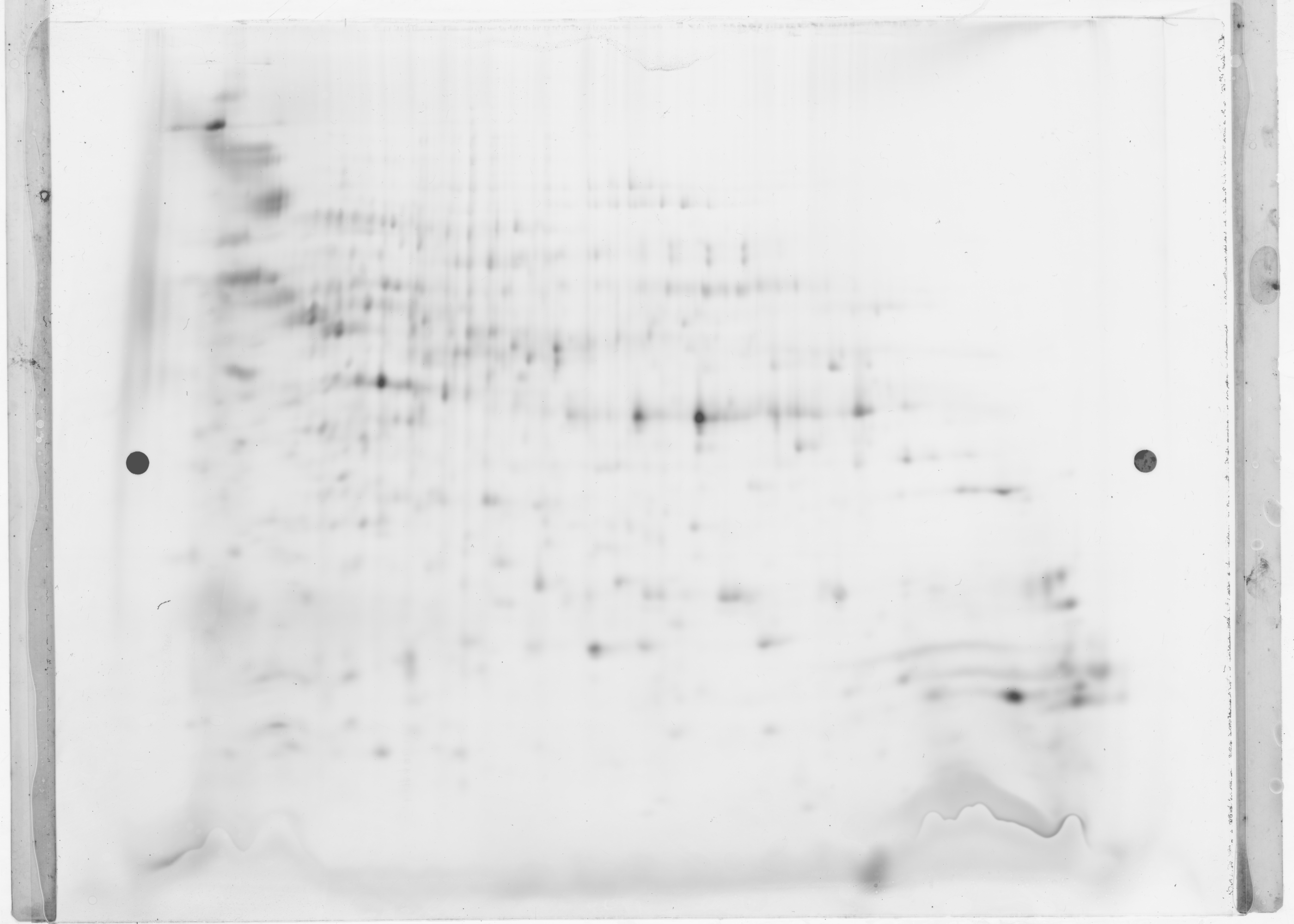

Supplement: Supplementary file 26 — Supplementary material [file mmc26.zip › mmc26.gel]

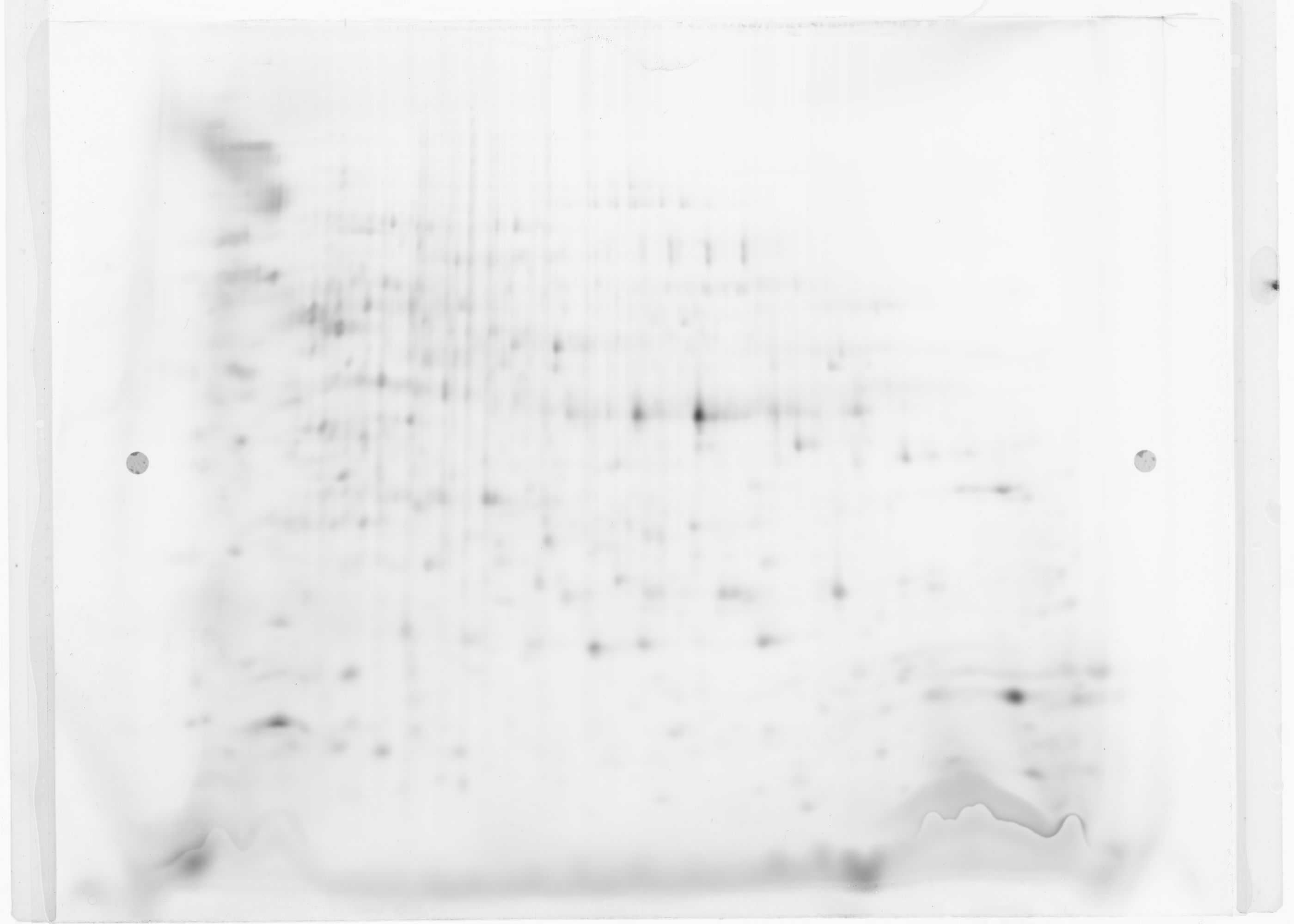

Supplement: Supplementary file 27 — Supplementary material [file mmc27.zip › mmc27.gel]

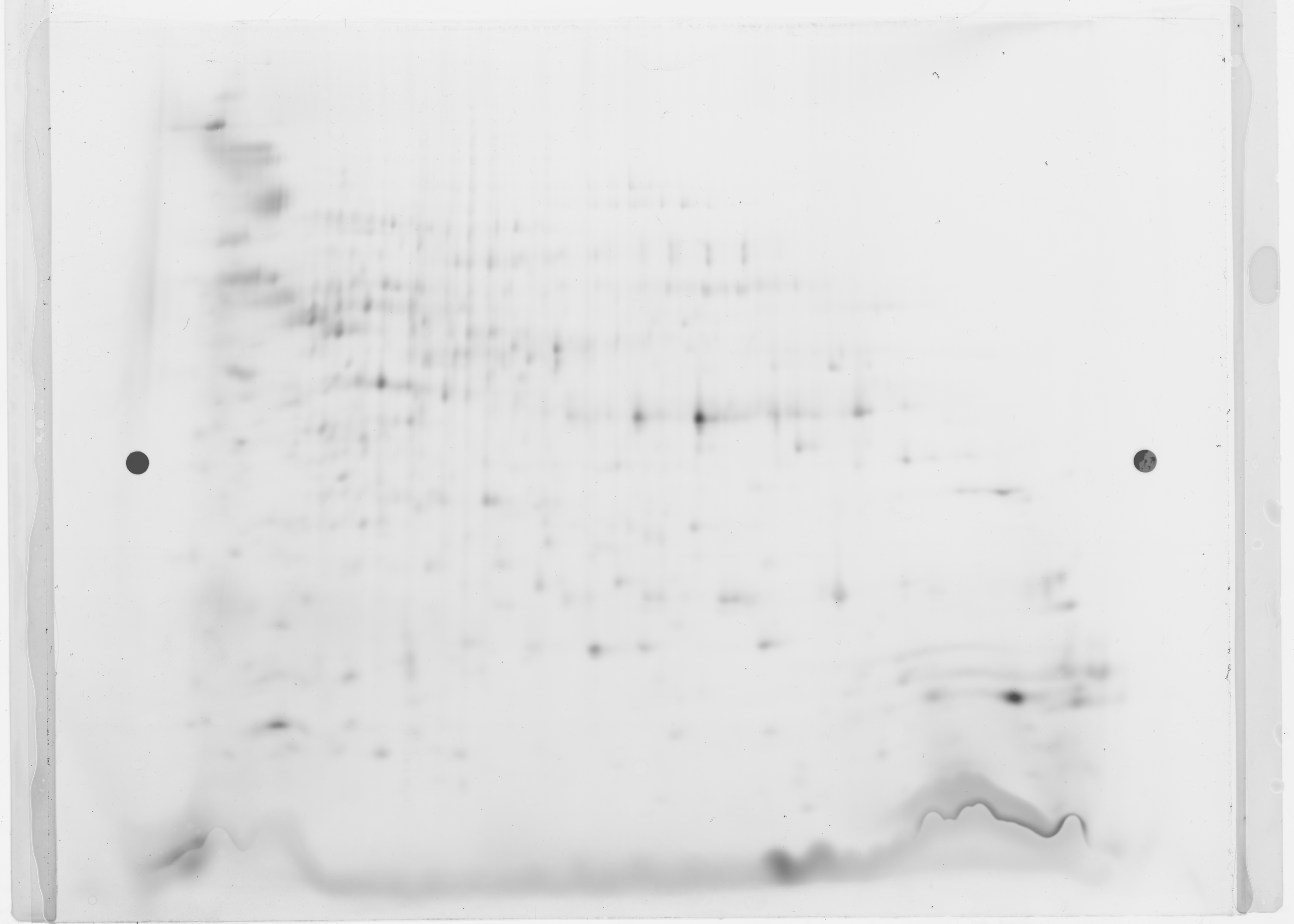

Supplement: Supplementary file 28 — Supplementary material [file mmc28.zip › mmc28.gel]

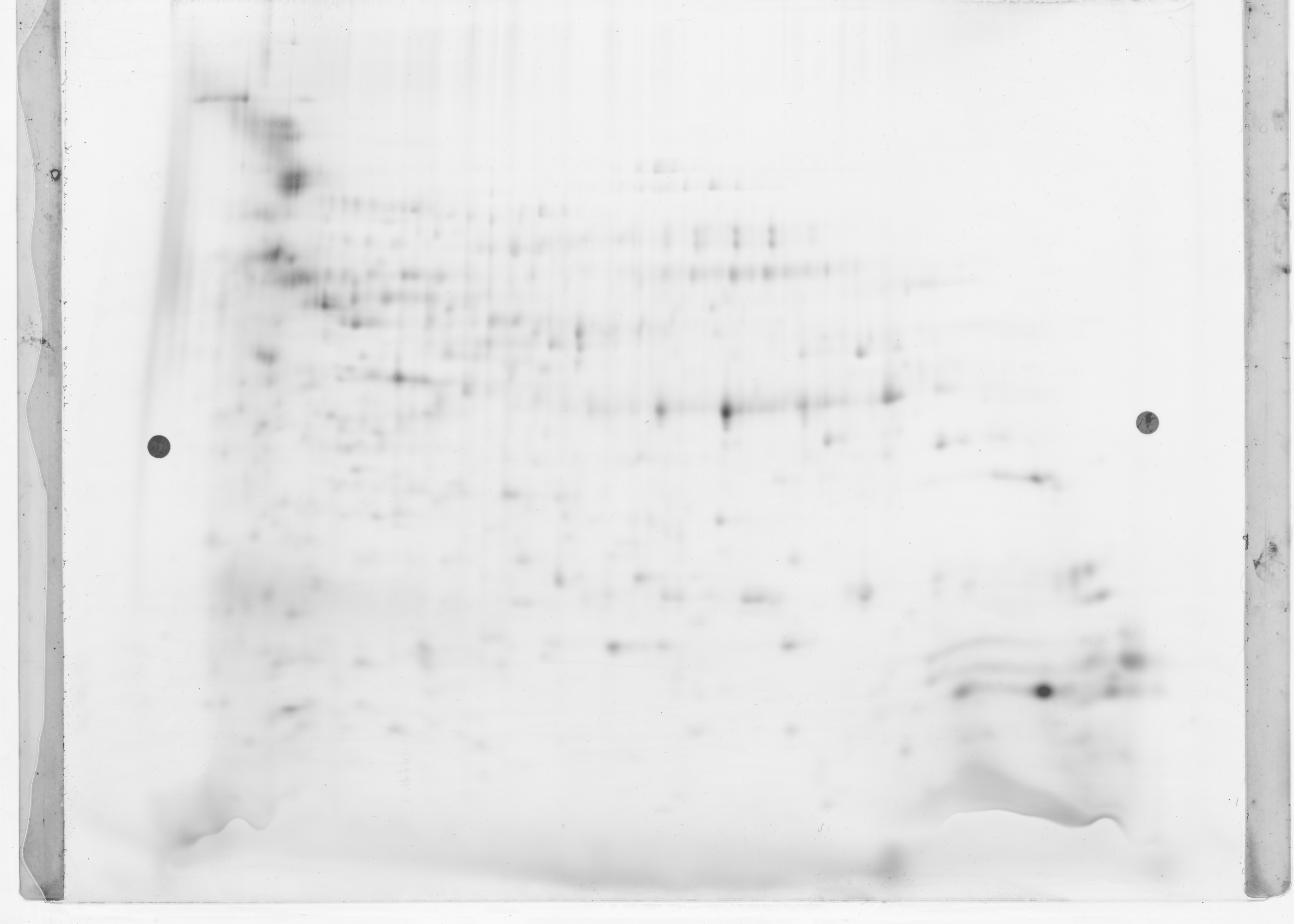

Supplement: Supplementary file 29 — Supplementary material [file mmc29.zip › mmc29.gel]

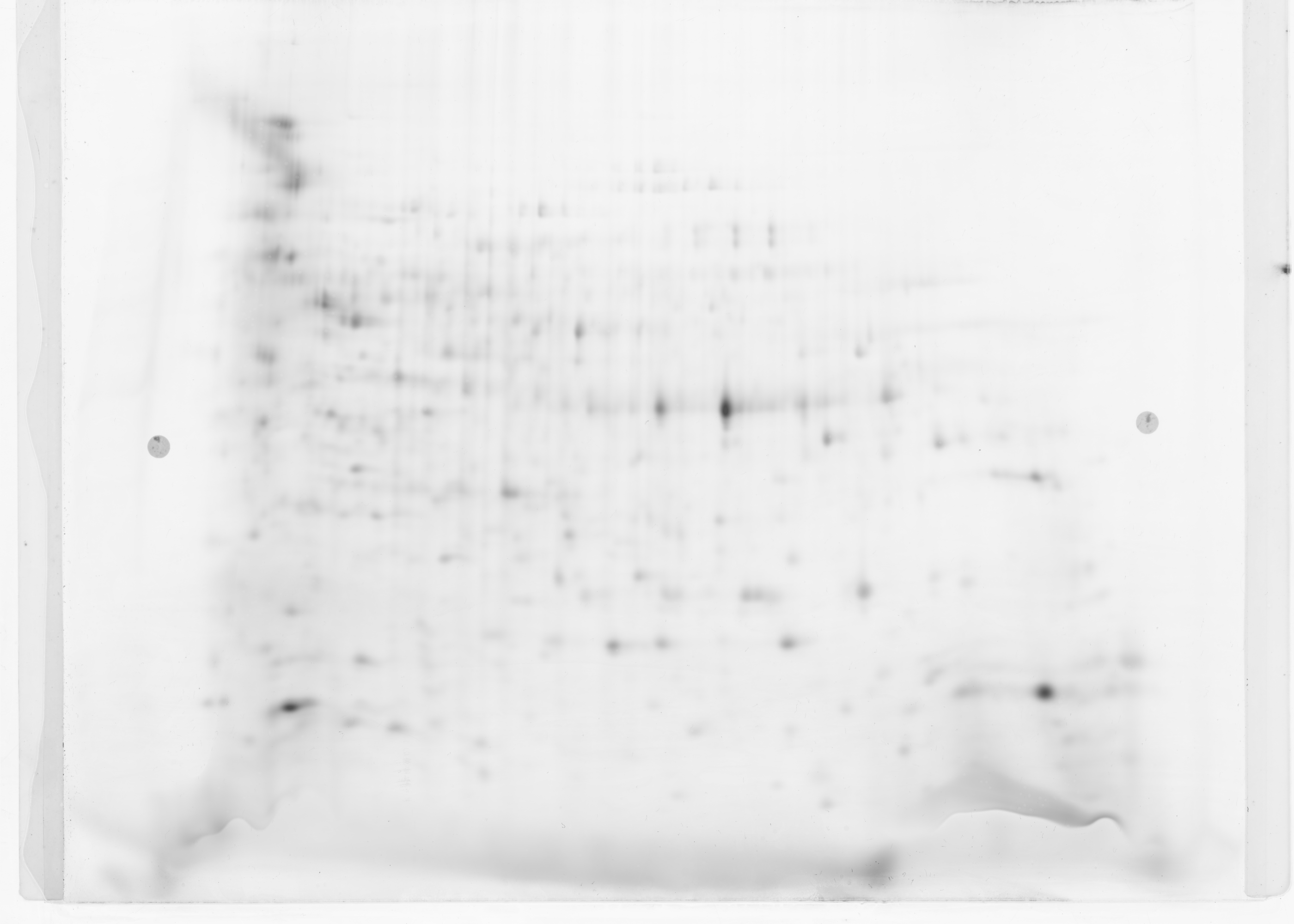

Supplement: Supplementary file 30 — Supplementary material [file mmc30.zip › mmc30.gel]

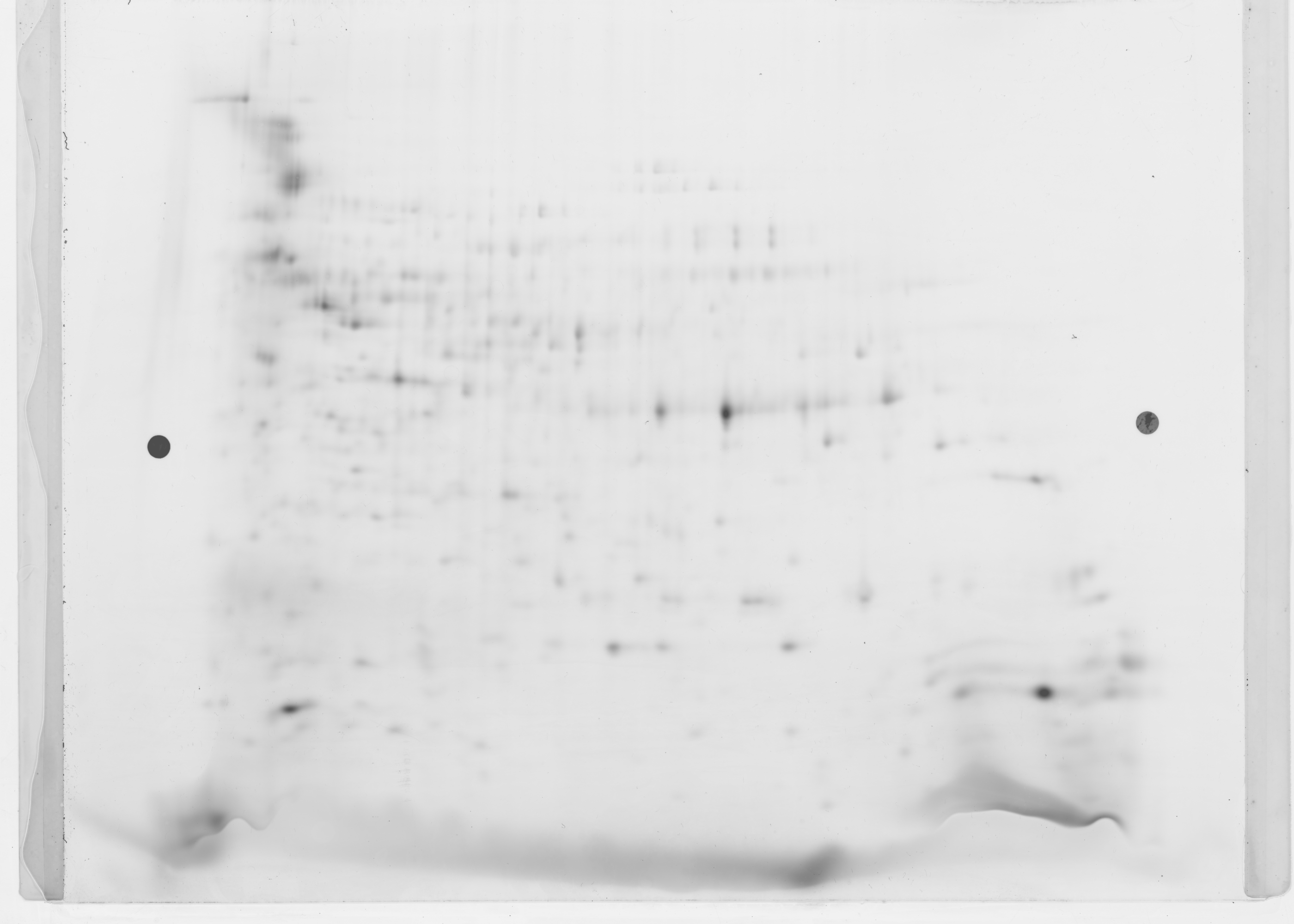

Supplement: Supplementary file 31 — Supplementary material [file mmc31.zip › mmc31.gel]

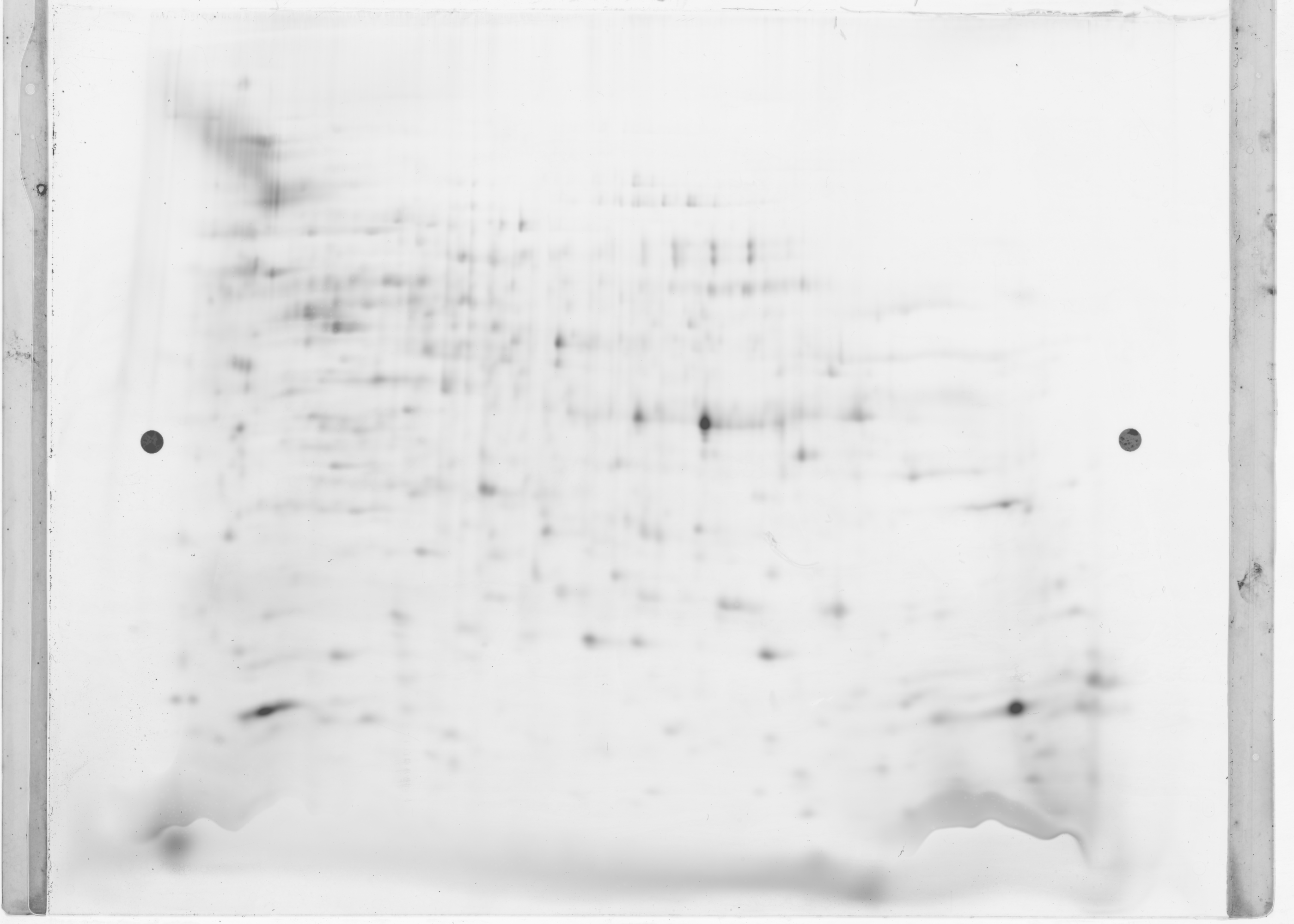

Supplement: Supplementary file 32 — Supplementary material [file mmc32.zip › mmc32.gel]

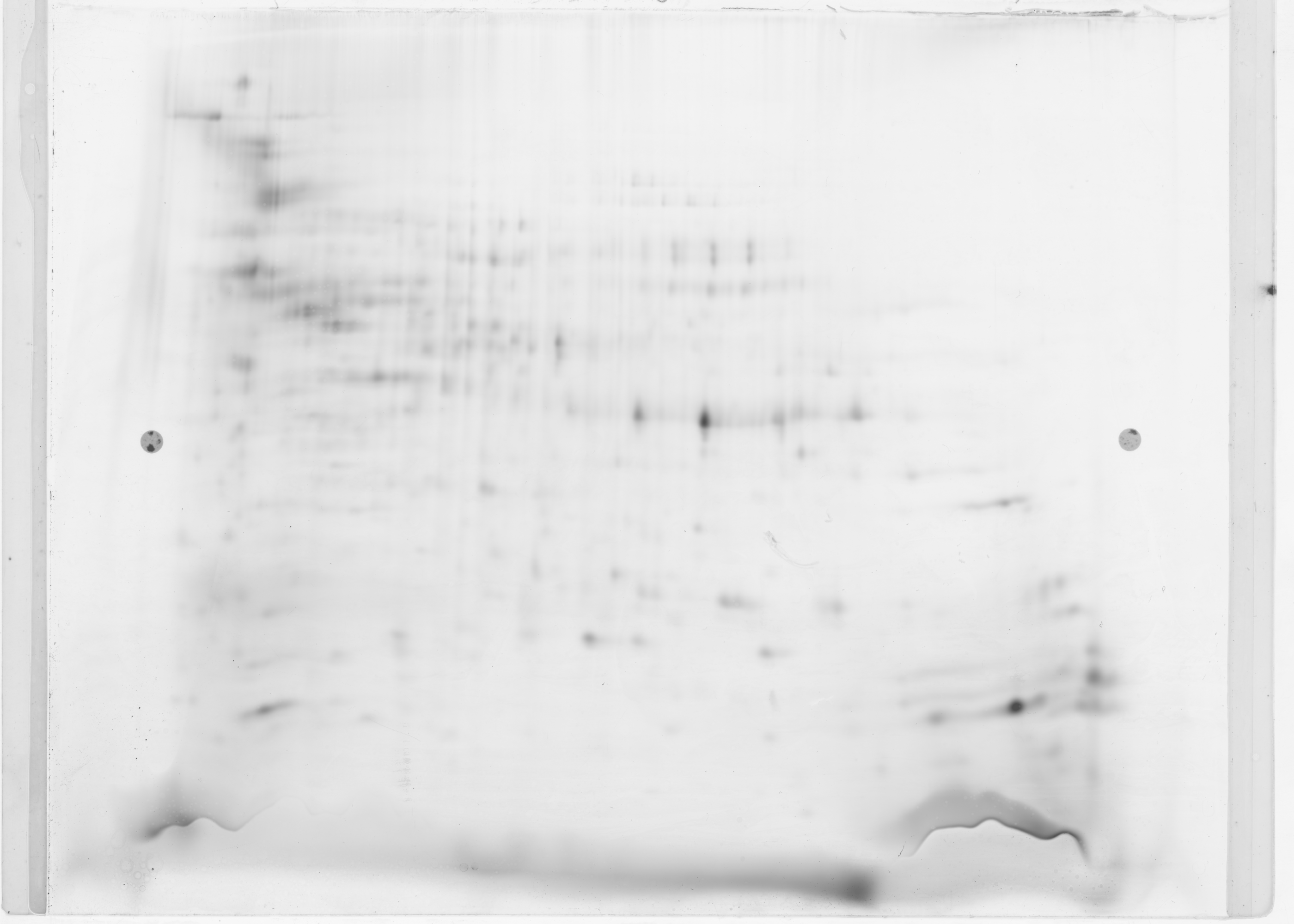

Supplement: Supplementary file 33 — Supplementary material [file mmc33.zip › mmc33.gel]

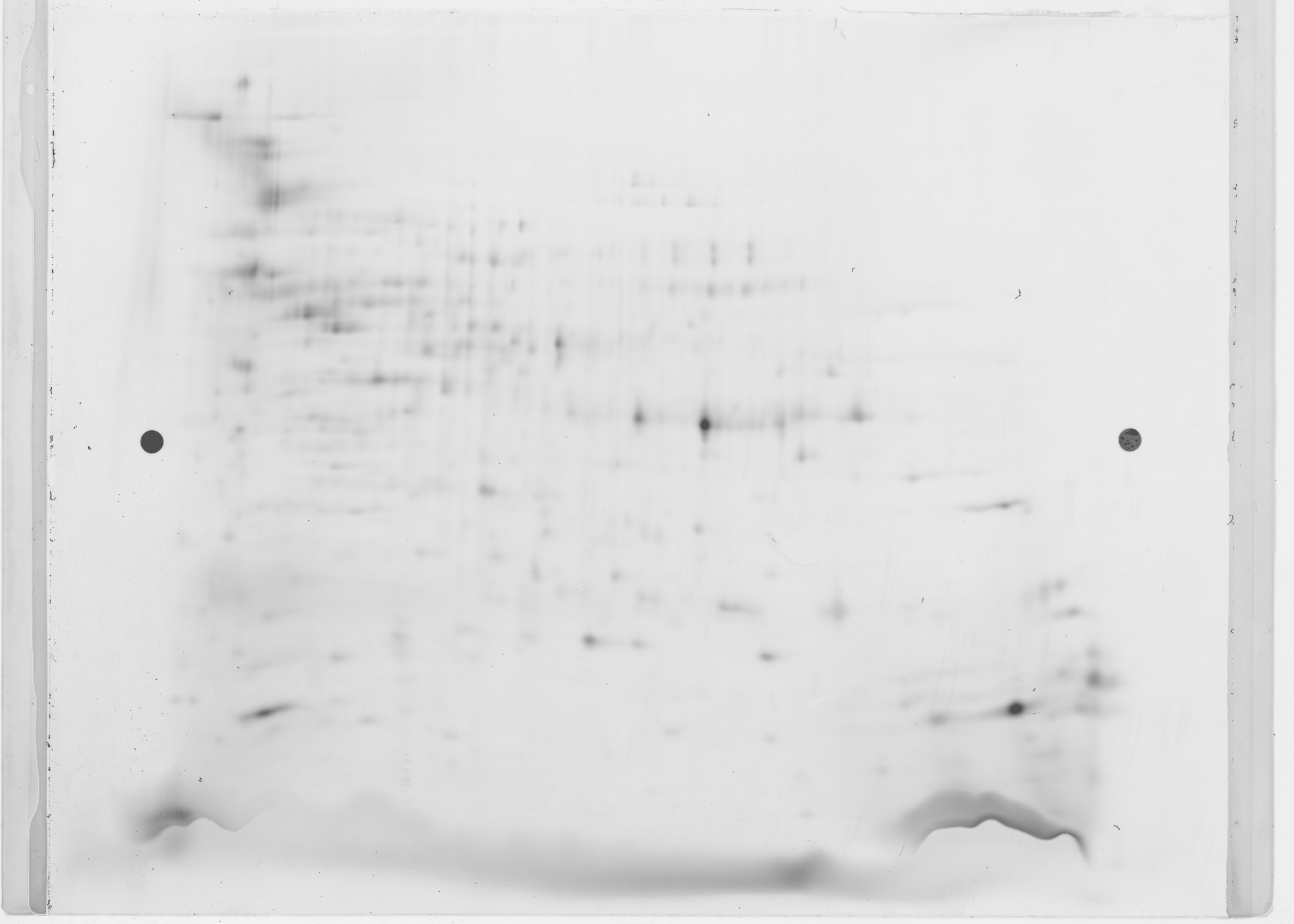

Supplement: Supplementary file 34 — Supplementary material [file mmc34.zip › mmc34.gel]

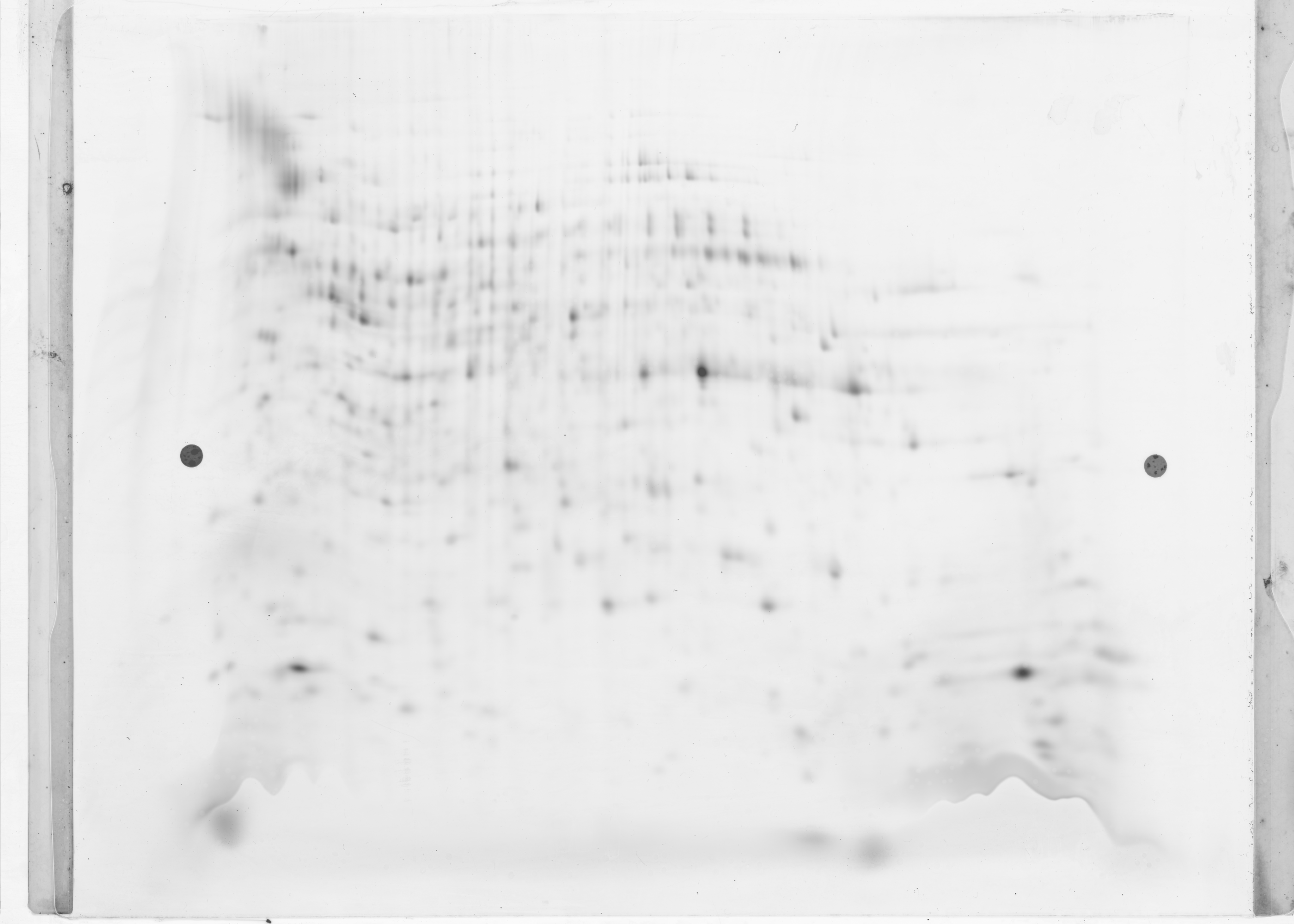

Supplement: Supplementary file 35 — Supplementary material [file mmc35.zip › mmc35.gel]

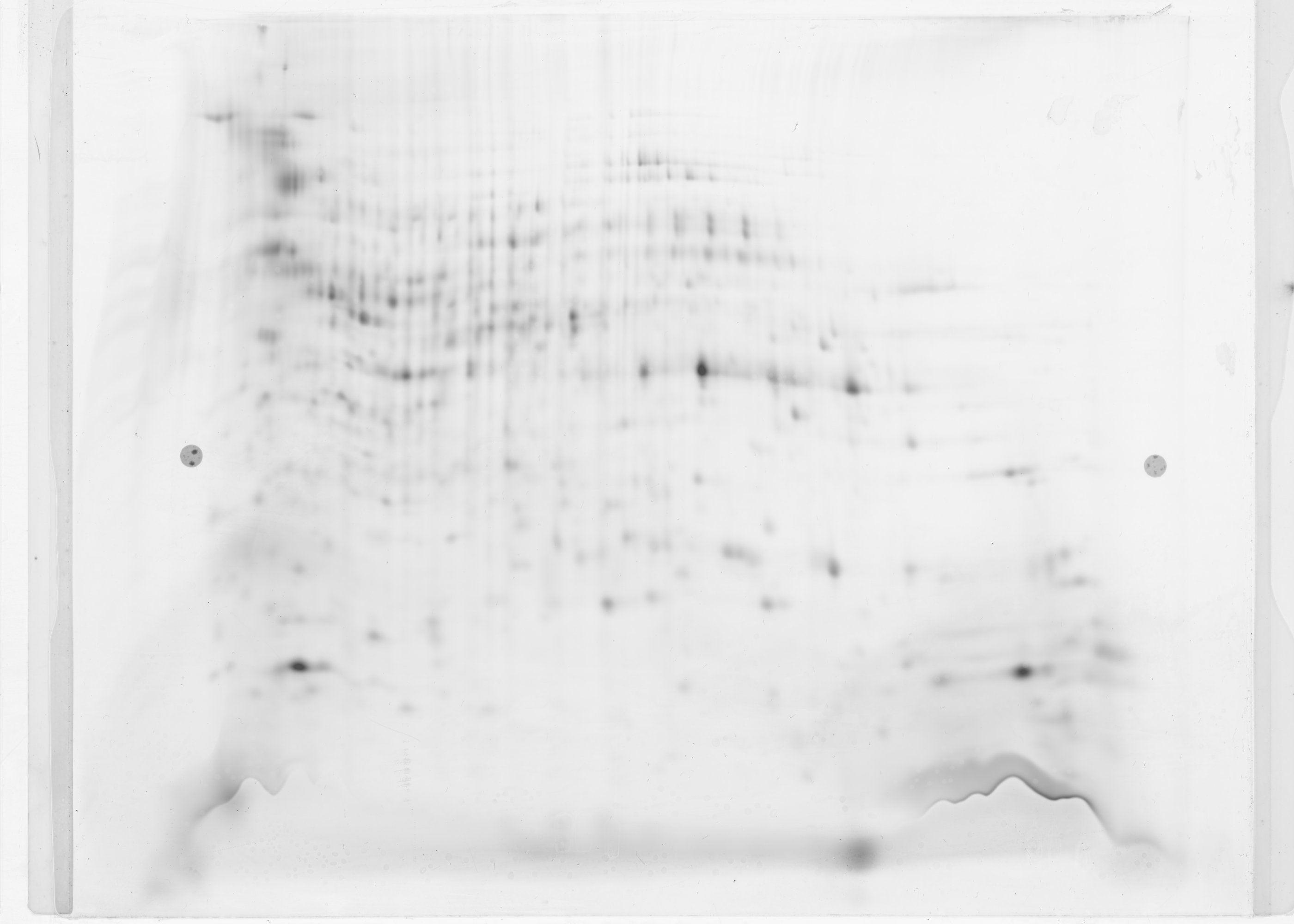

Supplement: Supplementary file 36 — Supplementary material [file mmc36.zip › mmc36.gel]

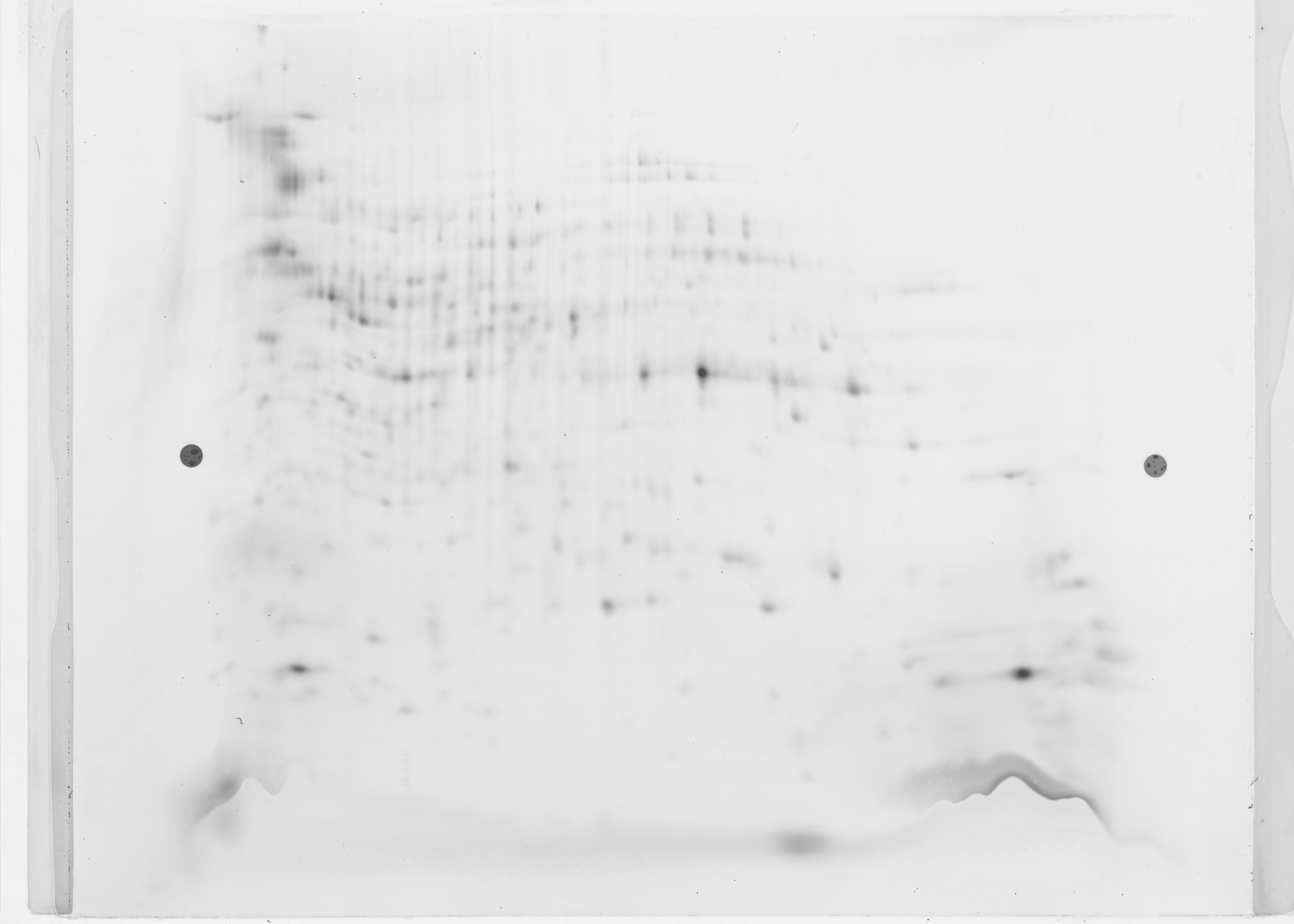

Supplement: Supplementary file 37 — Supplementary material [file mmc37.zip › mmc37.gel]
